# Polydopamine‐Encapsulated Probiotics Restore Gut Homeostasis and Reinstate Macrophage Efferocytosis in Systemic Lupus Erythematosus

**DOI:** 10.1002/advs.202522637

**Published:** 2026-02-20

**Authors:** Ruimiao Wu, Mei Liu, Changxing Gao, Cheng Zhao, Feijie Zhao, Shuqing Dong, Bo Zhang, Qianmei Liu, Wenqian Zhang, Ming Zhao, Qianjin Lu

**Affiliations:** ^1^ Hospital for Skin Diseases, Institute of Dermatology Chinese Academy of Medical Sciences and Peking Union Medical College Nanjing China; ^2^ Key Laboratory of Basic and Translational Research On Immune‐Mediated Skin Diseases Chinese Academy of Medical Sciences Nanjing China; ^3^ Jiangsu Provincial Key Laboratory of Dermatology Nanjing China; ^4^ Shandong First Medical University Jinan China; ^5^ Hunan University of Technology Zhuzhou China; ^6^ Department of Dermatology, Hunan Key Laboratory of Medical Epigenomics the Second Xiangya Hospital, Central South University Changsha China

**Keywords:** CX3CR1, efferocytosis, gut microbiota, *lactobacillus rhamnosus GG*, L‐methionine, polydopamine, systemic lupus erythematosus (SLE)

## Abstract

Systemic lupus erythematosus (SLE) is an autoimmune disease characterized by immune dysregulation and chronic inflammation, with increasing evidence implicating the gut microbiota in its pathogenesis. Probiotics, such as *Lactobacillus rhamnosus GG* (LGG), exert anti‐inflammatory effects by enhancing gut barrier function and restoring microbial homeostasis, representing a promising therapeutic strategy for SLE. However, conventional probiotic therapies are hindered by poor survival and colonization in the hostile intestinal environment. Here, a polydopamine‐coated LGG (LGG@PDA) with improved viability, adhesion, and resistance to oxidative stress is developed. In murine models of lupus, LGG@PDA treatment restored gut and immune homeostasis, enhanced macrophage efferocytosis, reduced autoantibody levels, and ameliorated renal pathology. Metabolomic analysis further identified L‐methionine, a metabolite diminished in both lupus mice and SLE patients, as being enriched by LGG@PDA treatment. Functionally, L‐methionine enhanced macrophage efferocytosis in a CX3CR1‐dependent manner, thereby contributing to the restoration of immune tolerance. Collectively, these findings establish LGG@PDA as a bioengineered probiotic platform that integrates microbiota modulation with immune regulation, highlighting L‐methionine as a key metabolic mediator and a promising microbiota‐based therapeutic strategy for SLE.

## Graphical abstract

Polydopamine‐encapsulated probiotics restore gut homeostasis and reinstate macrophage efferocytosis, thereby reestablishing immune tolerance and mitigating systemic autoimmunity, highlighting a bioengineered microbiota‐based therapeutic strategy for SLE.

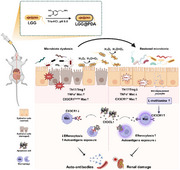

## 1 Introduction

Systemic lupus erythematosus (SLE) is a chronic, relapsing autoimmune disease characterized by multisystem involvement, excessive autoantibody production, and dysregulated immune activation [1]. Notably, aberrant increases in apoptosis, coupled with the insufficient clearance of late apoptotic cellular debris by macrophages has been identified as key drivers of self‐tolerance breakdown and autoimmunity initiation [2]. Inefficient clearance of apoptotic cells leads to the accumulation of secondary necrotic cells, which release nuclear autoantigens and damage‐associated molecular patterns (DAMPs) that subsequently activate antigen‐presenting cells and trigger type I interferon responses, ultimately promoting immune dysregulation in lupus [3, 4]. Despite therapeutic advances, current treatments primarily rely on corticosteroids and broad‐spectrum immunosuppressants, which effectively control acute flares but often lead to cumulative toxicity and heightened infections risk with prolonged use [5]. These limitations underscore the urgent need for novel strategies to restore immune homeostasis without compromising host defense.

Emerging evidence underscores the pivotal role of gut microbiota in modulating immune tolerance and systemic inflammation [6, 7]. Dysbiosis, characterized by reduced microbial diversity and enrichment of pro‐inflammatory taxa, is consistently observed in SLE patients and lupus‐prone mice [8, 9, 10, 11, 12]. Notably, fecal microbiota transfer from patients into germ‐free mice has been shown to induce lupus‐like phenotypes in the recipients [13]. Such dysbiosis may disrupt barrier integrity and alter metabolic output, thereby sustaining systemic immune activation and impairing immune tolerance [14]. Conversely, interventions such as fecal microbiota transplantation or probiotic administration have been shown to ameliorate lupus manifestations in both patients and animal models, suggesting that modulation of the gut ecosystem represents a promising therapeutic strategy [15, 16].

Probiotics, especially *Lactobacillus* and *Bifidobacterium*, exert anti‐inflammatory effects by their intrinsic bioactive components and metabolites, which may reinforce the intestinal barrier, attenuate inflammatory responses, and restore gut microbial homeostasis [17, 18]. Among them, accumulating evidence suggests that *Lactobacillus rhamnosus GG* (LGG), a clinically used probiotic with proven safety and immunomodulatory properties, exerts particularly promising therapeutic effects in SLE. In lupus‐prone mice, LGG supplementation or *Lactobacillus* consortia restore gut microbial balance, improve renal pathology, and rebalance Th17/Treg responses [19, 20, 21, 22]. In vitro, LGG promotes tolerogenic dendritic cells, skews macrophages toward anti‐inflammatory phenotypes, and modulates microRNA and cytokine expression in SLE patient‐derived immune cells [23, 24, 25, 26, 27]. Clinical trials suggest that synbiotic or probiotic supplementation containing LGG can reduce inflammatory mediators and disease activity, supporting its potential as an adjunctive strategy in SLE [28, 29]. However, their therapeutic efficacy remains constrained by poor survival in the harsh gastrointestinal environment, rapid clearance and impaired colonization under inflammatory and oxidative conditions.

To address these challenges, material‐assisted probiotic delivery systems have emerged as a promising strategy. Polydopamine (PDA), a bioinspired polymer with excellent biocompatibility, pH responsiveness, and antioxidative properties, offers advantages for bacterial protection and controlled release [30, 31]. Here, a PDA‐coated LGG formulation (LGG@PDA) was engineered to enhance bacterial survival, mitigate oxidative stress, and sustain intestinal colonization. We found that LGG@PDA administration in murine models of lupus significantly restored gut and immune homeostasis, and elevated L‐methionine levels, which in turn enhanced macrophage efferocytosis, and ultimately alleviated lupus‐like phenotypes.

## 2 Results

### 2.1 Preparation and Characterization of LGG@PDA

**FIGURE 1:**
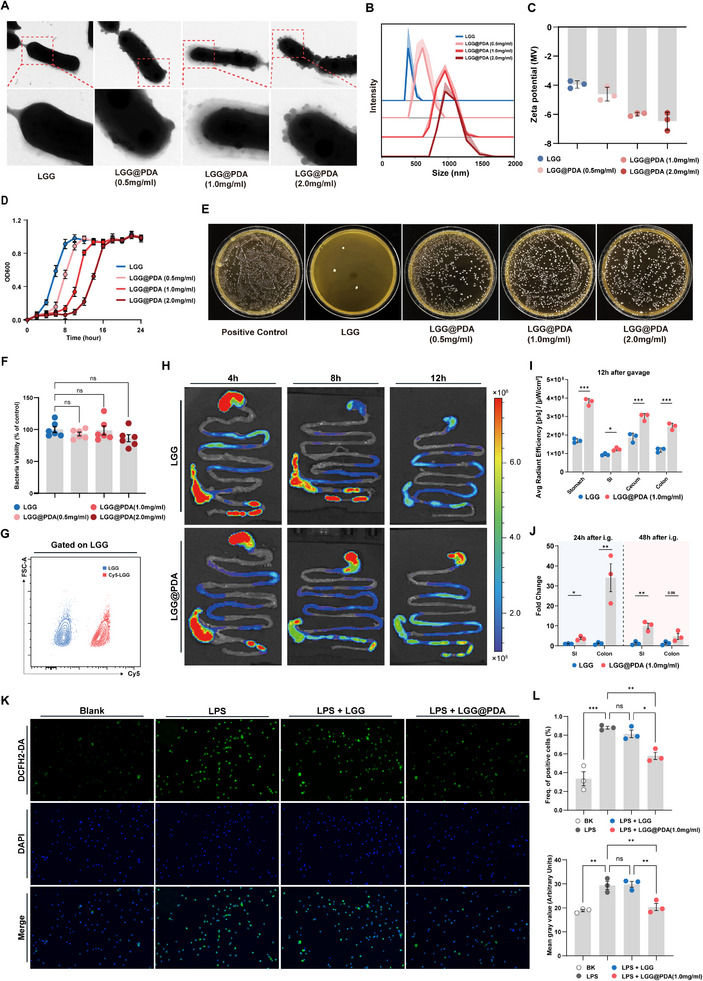
Preparation and functional characterization of LGG@PDA. (A–C) Transmission electron microscopy (TEM) images, particle size distribution (DLS), and zeta potential analysis of LGG coated with different concentrations of PDA (0, 0.5, 1.0, and 2.0 mg mL^−1^). (D) Growth curves of LGG and LGG@PDA at various PDA concentrations. (E) In vitro gastrointestinal tolerance assay following sequential exposure to simulated gastric and intestinal fluids. (F) CCK‐8 assays of log‐phase cultures of LGG and LGG@PDA. (G) Flow cytometry analysis validating Cy5 labeling efficiency of LGG and LGG@PDA. (H‐I) Fluorescence imaging of gastrointestinal tissues following oral administration of Cy5‐labeled LGG or LGG@PDA at 4, 8, and 12 h, with quantitative analysis at 12 h. (J) Quantitative PCR analysis of intestinal contents at 24 and 48 h to assess bacterial abundance. (K, L) measurement of intracellular ROS levels in macrophages co‐cultured with LGG or LGG@PDA using the DCFH2‐DA probe. Data are presented as the mean ± SD. Statistical significance was determined using unpaired Student's *t*‐test (C, I, J) and one‐way ANOVA (F, L).  ^∗^ *p* < 0.05, ^∗∗^ *p* < 0.01, ^∗∗∗^ *p* < 0.001, ^∗∗∗∗^ *p* < 0.0001; ns, not significant.

To establish a bioengineered probiotic platform, LGG was coated with varying concentrations of dopamine hydrochloride (0.5, 1.0, and 2.0 mg mL^−1^) under alkaline conditions (pH 8.8). Transmission electron microscopy (TEM) showed that LGG exhibited a uniform and continuous PDA layer at 1.0 mg mL^−1^ of dopamine hydrochloride, whereas excessive PDA induced self‐polymerization into particulate aggregates adhering to LGG (Figure 1A). Dynamic light scattering (DLS) analysis demonstrated a concentration‐dependent enlargement of LGG@PDA particles, with mean diameters of 619.3, 911.7, and 1006.7 nm at 0.5, 1.0 and 2.0 mg mL^−1^, respectively (Figure 1B). Consistently, zeta potential measurements indicated a progressive surface charge shift, confirming successful synthesis of PDA capsule deposition (Figure 1C). Collectively, these findings indicate that 1.0 mg mL^−1^ dopamine hydrochloride offers optimal coating homogeneity and physicochemical stability.

The influence of PDA encapsulation on bacterial physiology was then assessed. As shown in Figure 1D, a slight delay was observed in the initial growth rate of LGG as the concentration of PDA increased, whereas the exponential growth phase was unaffected (Figure 1D). Moreover, CCK‐8 assays of log‐phase cultures confirmed that PDA coating did not compromise bacterial viability (Figure 1F). We next investigated whether PDA encapsulation could enhance the resilience of LGG under harsh gastrointestinal conditions. In vitro tolerance assays were conducted by sequentially incubating LGG and LGG@PDA in simulated gastric fluid for 4 h followed by simulated intestinal fluid for 2 h, after which bacterial viability was quantified by colony‐forming unit (CFU) enumeration. The results revealed a pronounced enhancement of survival in LGG@PDA, with 1.0 mg mL^−1^ of dopamine hydrochloride formulation conferring the most significant protection (Figure 1E). Based on these findings, the 1.0 mg mL^−1^ of dopamine hydrochloride formulation was selected for subsequent functional investigations.

To further evaluate gastrointestinal retention in vivo, LGG was labeled with Cy5 (Figure 1G). Equal doses of Cy5‐LGG and Cy5‐LGG@PDA were orally administered to mice, and the distribution of the labeled bacteria was monitored using an in vivo imaging system (IVIS). Free Cy5‐LGG exhibited rapid clearance, whereas Cy5‐LGG@PDA displayed markedly prolonged retention throughout the gastrointestinal tract (Figure 1H). At 12 h post‐administration, fluorescence signals in the stomach, small intestine, cecum, and colon were significantly stronger in the LGG@PDA group compared to the uncoated LGG group (Figure 1I). To further evaluate the colonization and proliferation of LGG, quantitative real‐time PCR (qPCR) analysis of intestinal contents at 24 and 48 h was performed (Figure 1J). Consistently, significantly higher bacterial abundance was observed in the mice receiving LGG@PDA. These results further validated the improved gastrointestinal persistence conferred by PDA encapsulation.

Finally, the antioxidant activity of LGG@PDA was evaluated. Macrophages were co‐cultured with LGG@PDA, and intracellular reactive oxygen species (ROS) levels were assessed using the fluorescent probe 2',7'‐ dichlorodihydrofluorescein diacetate (DCFH_2_‐DA). The results indicated that LGG@PDA efficiently scavenged intracellular ROS levels (Figure 1K,L), underscoring its dual functionality as a probiotic delivery platform and an active modulator of oxidative stress.

### 2.2 Potential of the LGG@PDA for the Treatment of SLE

**FIGURE 2:**
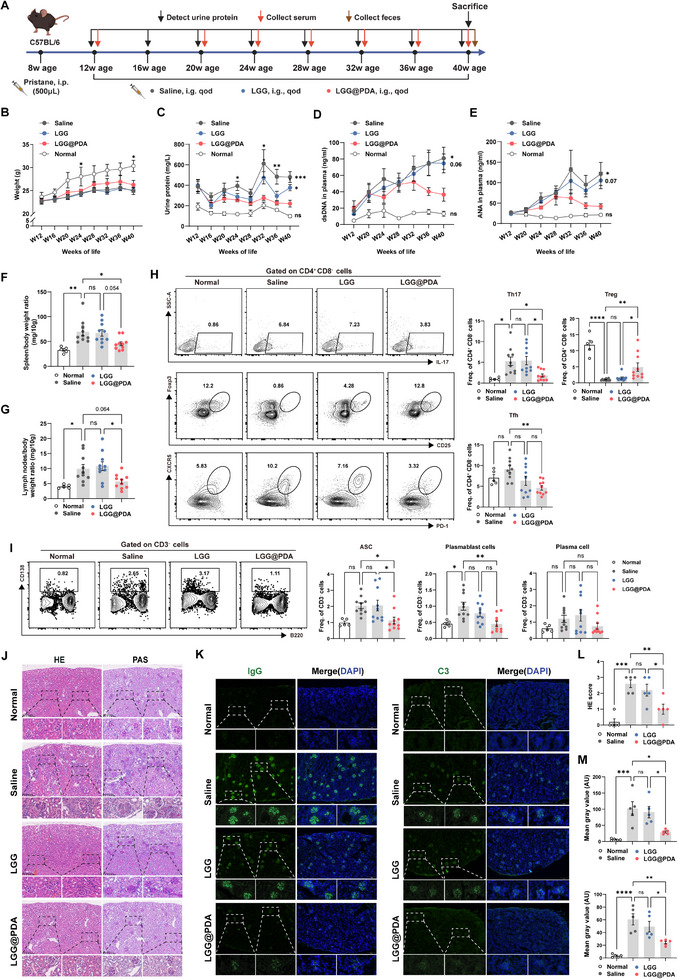
Therapeutic efficacy of LGG@PDA in pristane‐induced lupus‐like mice. (A) Schematic overview of the experimental design. Lupus was induced in mice by intraperitoneal injection of pristane, followed by random allocation to receive oral saline, free LGG, or LGG@PDA (*n* = 10 per group), alongside a normal control group (*n* = 5). (B–E) Longitudinal monitoring of disease progression over 28 weeks, including body weight (B), urinary protein excretion (C), and plasma levels of anti‐dsDNA antibodies (D) and antinuclear antibodies (E). (F,G) Spleen and lymph node weights normalized to body weight at the experimental endpoint. (H,I) Flow cytometry analysis of splenic T and B cell subsets, including Th17 (Zombie Aqua^−^CD3^+^CD4^+^CD8^−^IL17A^+^), Tfh (Zombie Aqua^−^CD3^+^CD4^+^CD8^−^CXCR5^+^PD‐1^+^), Treg (Zombie Aqua^−^CD3^+^CD4^+^CD8^−^CD25^+^Foxp3^+^), antibody‐secreting B cells (Zombie Aqua^−^CD3^−^CD138^+^), plasmablasts (Zombie Aqua^−^CD3^−^B220^+^CD138^+^) and plasma cells (Zombie Aqua^−^CD3^−^B220^−^CD138^+^). (J,L) Representative H&E and PAS staining of kidney sections (J) and quantification of renal injury scores (L). (K,M) Immunofluorescence staining (K) and quantitative analysis of IgG (M, top) and C3(M, bottom) deposition in renal glomeruli. Each point represents one subject, and bars indicate the mean ± SEM. Statistical significance was determined using one‐way ANOVA (B‐sM).  ^∗^ *p* < 0.05, ^∗∗^ *p* < 0.01, ^∗∗∗^ *p* < 0.001, ^∗∗∗∗^ *p* < 0.0001; ns, not significant.

To evaluate the therapeutic potential of LGG@PDA, we first employed a pristane‐induced lupus‐like murine model, which recapitulates key features of human SLE, including proteinuria, elevated autoantibody levels, immune complex–mediated glomerulonephritis, and alterations in immune cell populations such as an imbalanced Th17/Treg ratio, an expanded Tfh cell response, and increased differentiation of B cell subsets responsible for autoantibody production [32, 33, 34, 35, 36]. Its efficacy was subsequently evaluated in MRL/lpr spontaneous lupus‐prone mice, confirming the therapeutic benefit in both induced and spontaneous lupus models. The experimental design and corresponding flowchart for the pristane model are presented in Figure 2A. Mice were randomly allocated to receive oral administration of saline, free LGG, or LGG@PDA, while age‐matched healthy mice served as normal controls. Longitudinal monitoring revealed that LGG@PDA significantly attenuated disease progression, as indicated by reduced urinary protein excretion (Figure 2C), serum anti‐dsDNA (Figure 2D) and ANA antibody levels (Figure 2E), approaching levels observed in healthy controls. Body weight remained comparable among SLE mice (Figure 2B), whereas spleen and lymph node weights relative to body weight were decreased in LGG@PDA‐treated mice, indicating alleviation of splenomegaly and lymphadenopathy (Figure 2F,G). To verify the underlying immunomodulatory effects of LGG@PDA, splenic T and B cell subsets were analyzed by flow cytometry. The results demonstrated that LGG@PDA administration significantly reduced the frequencies of Th17 cells, Tfh cells, antibody‐secreting B cells (ASCs) and plasmablasts, while promoting Treg differentiation (Figure 2H,I), indicating a shift toward an immunoregulatory phenotype. Other T and B cell subsets remained largely unchanged (Figure S1). Renal pathology, a defining feature of advanced SLE, was substantially improved, as H&E and PAS staining revealed reduced inflammatory infiltration and preserved glomerular architecture (Figure 2J), accompanied by lower renal injury scores (Figure 2L). Furthermore, immunofluorescence analysis revealed diminished IgG and C3 deposition in glomeruli, approaching levels observed in healthy controls (Figure 2K,M).

**FIGURE 3:**
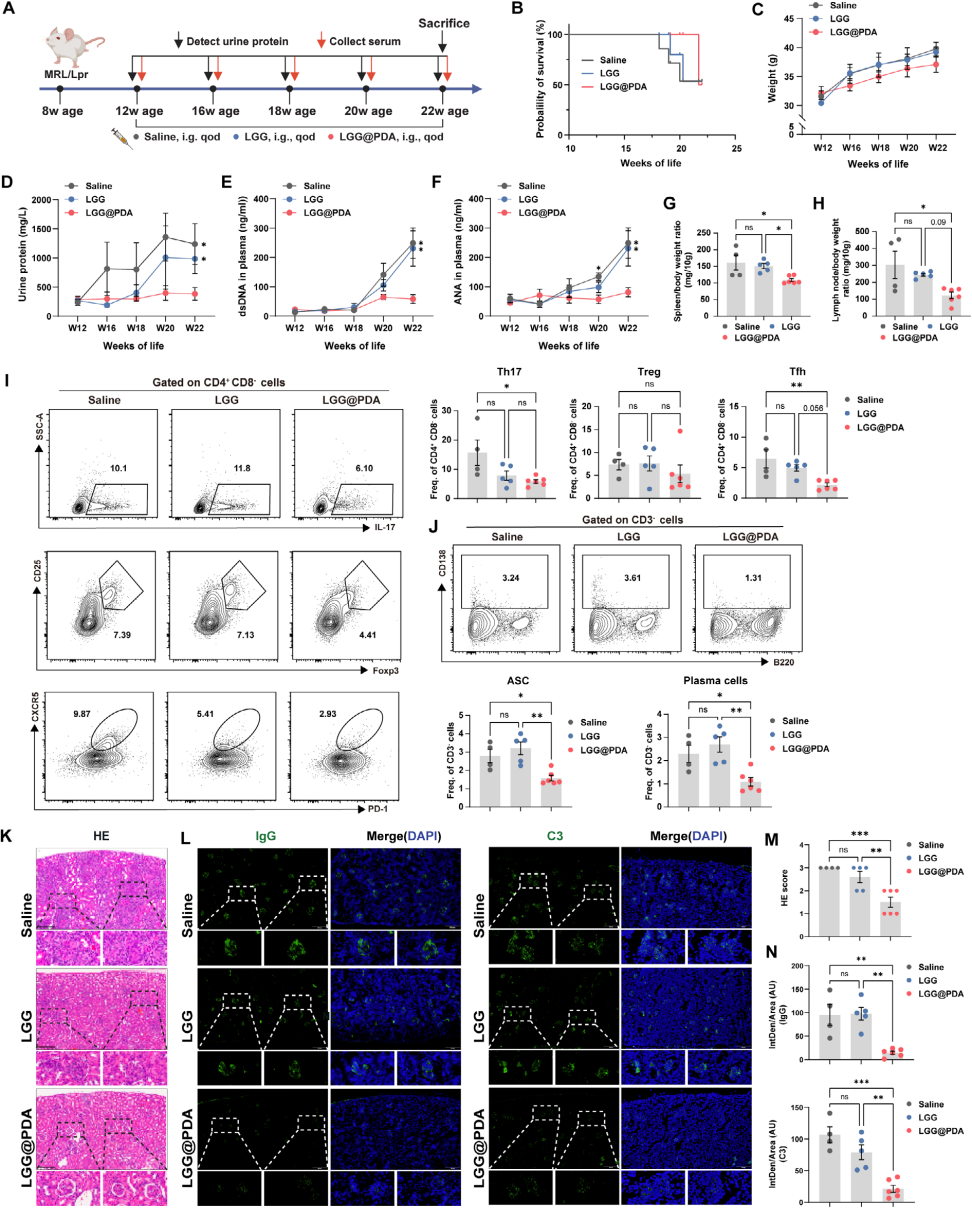
Therapeutic efficacy of LGG@PDA in *MRL/lpr* spontaneous lupus‐prone mice. (A) Schematic overview of the experimental design. Mice were randomly allocated to receive oral saline, free LGG, or LGG@PDA (starting *n* = 7 per group). (B) Survival curves of *MRL/lpr* mice in each treatment group. (C–F) Longitudinal monitoring of disease progression, including body weight (C), urinary protein excretion (D), and levels of anti‐dsDNA antibodies (E) and antinuclear antibodies (F). (G,H) Spleen and lymph node weights normalized to body weight at the experimental endpoint. (I,J) Flow cytometry analysis of splenic T and B cell subsets, including Th17 (Zombie Aqua^−^CD3^+^CD4^+^CD8^−^IL17A^+^), Tfh (Zombie Aqua^−^CD3^+^CD4^+^CD8^−^CXCR5^+^PD‐1^+^), Treg (Zombie Aqua^−^CD3^+^CD4^+^CD8^−^CD25^+^Foxp3^+^), antibody‐secreting B cells (Zombie Aqua^−^CD3^−^CD138^+^), and plasma cells (Zombie Aqua^−^CD3^−^B220^−^CD138^+^). (K,M) Representative H&E staining of kidney sections (K) and quantification of renal injury scores (M). (L,N) Immunofluorescence staining (L) and quantitative analysis of IgG (N, top) and C3(N, bottom) deposition in renal glomeruli. Each point represents one subject, and bars indicate the mean ± SEM. Statistical significance was determined using one‐way ANOVA (C–N).  ^∗^ *p* < 0.05, ^∗∗^ *p* < 0.01, ^∗∗∗^ *p* < 0.001, ^∗∗∗∗^ *p* < 0.0001; ns, not significant.

To further validate these findings in a chronic, spontaneous model, *MRL/lpr* lupus‐prone mice were examined (Figure 3A). LGG@PDA treatment improved survival relative to controls receiving saline or unmodified LGG (Figure 3B). A mild increase in body weight was observed in control groups, potentially reflecting lymphadenopathy at advanced disease stages (Figure 3C). Mice treated with LGG@PDA exhibited reductions in urinary protein excretion (Figure 3D), anti‐dsDNA antibody levels (Figure 3E) and ANA titers (Figure 3F). Relative to body weight, both spleen and lymph node weights were decreased, indicating mitigation of splenomegaly and lymphadenopathy (Figure 3G,H). Flow cytometric analysis of spleen revealed significant reductions in Th17 cells and Tfh cells frequencies, whereas Treg proportions remained largely unchanged (Figure 3I). ASC and plasma cell frequencies were also decreased (Figure 3J). Histopathological assessment of the kidney demonstrated preservation of glomerular architecture, attenuated inflammatory infiltration, and lower renal pathology scores (Figure 3K,M). Deposition of IgG and C3 in glomeruli was similarly diminished (Figure 3L,N), suggesting that the therapeutic effects of LGG@PDA in *MRL/lpr* mice closely mirror those observed in the pristane‐induced lupus model.

Collectively, these results demonstrate that LGG@PDA mediates robust immunomodulatory and renoprotective effects in both induced and spontaneous lupus models. It restores systemic immune homeostasis, suppresses renal immune complex deposition, and preserves kidney function, thereby supporting its potential as a promising therapeutic strategy for SLE.

### 2.3 LGG@PDA Alleviates Intestinal Oxidative Stress, Rebalances Mucosal Immune Responses, and Restores Barrier Integrity in Lupus Mice

**FIGURE 4:**
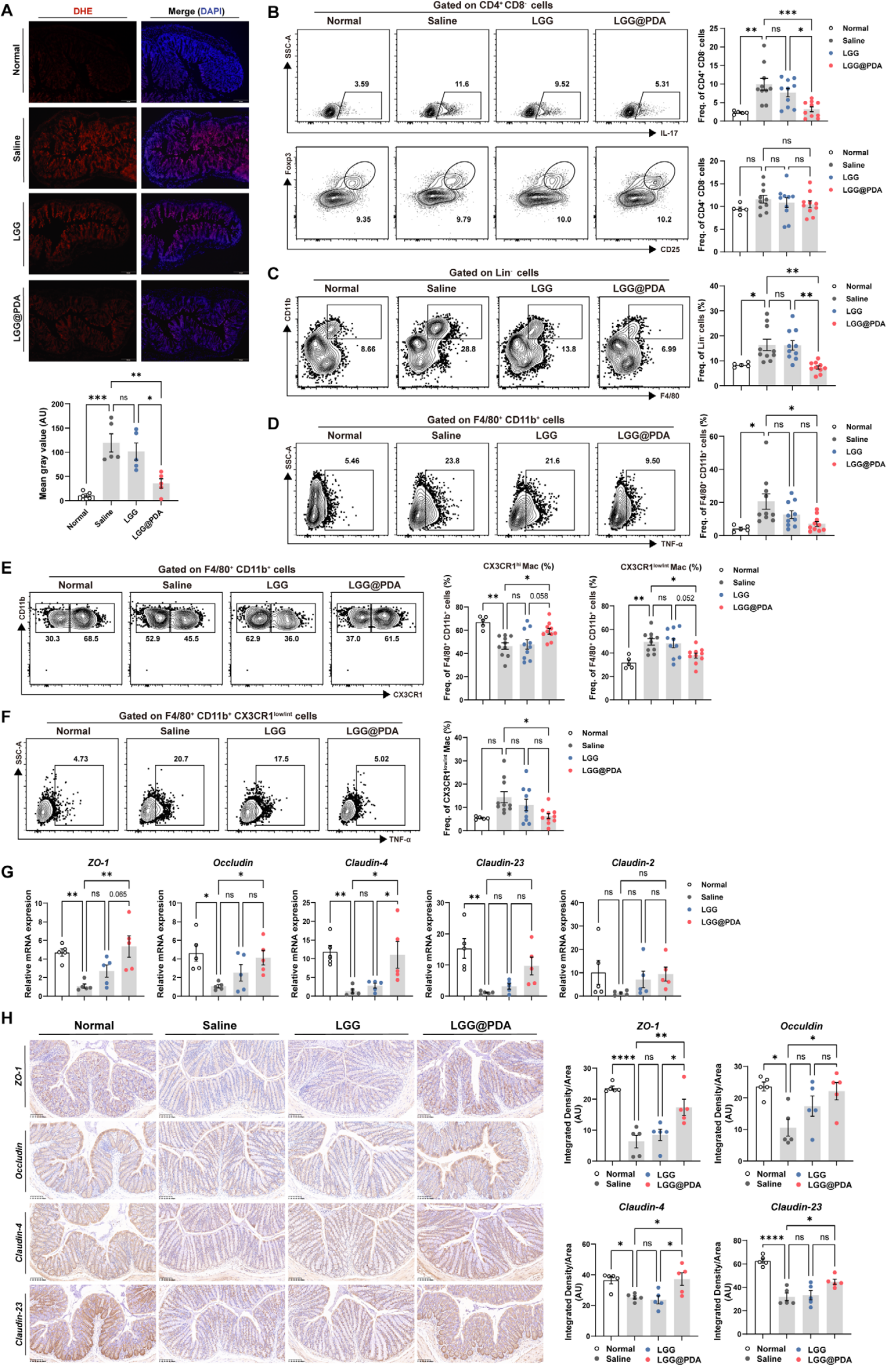
LGG@PDA reduces intestinal oxidative stress and restores mucosal immune balance. (A) Immunofluorescence staining of intestinal tissues for reactive oxygen species (ROS). (B) Flow cytometric analysis of T cell subsets in lamina propria, including Th17 (Zombie Aqua^−^CD3^+^CD4^+^CD8^−^IL17A^+^) and Treg cells (Zombie Aqua^−^CD3^+^CD4^+^CD8^−^CD25^+^Foxp3^+^). (C–F) Flow cytometric analysis of lamina propria macrophages, including total macrophages (C, Zombie Aqua^−^CD45^+^Lin^−^F4/80^+^CD11b^+^), TNF‐α^+^ macrophages (D, Zombie Aqua^−^CD45^+^Lin^−^F4/80^+^CD11b^+^TNF‐α^+^), CX3CR1^low/int^ and CX3CR1^hi^ macrophage subsets (E, Zombie Aqua^−^CD45^+^Lin^−^F4/80^+^CD11b^+^CX3CR1^low/int/hi^), and TNF‐α^+^ CX3CR1^low/int^ macrophages (F, Zombie Aqua^−^CD45^+^Lin^−^F4/80^+^CD11b^+^CX3CR1^low/int^TNF‐α^+^). (G) Quantitative PCR analysis of tight junction–related genes (*Zo‐1, Occludin, Claudin‐2, Claudin‐4, Claudin‐23*) in intestinal tissues. (H) Immunohistochemical staining and quantification of tight junction proteins. Each point represents one subject, and bars indicate the mean ± SEM. Statistical significance was determined using one‐way ANOVA.  ^∗^ *p* < 0.05, ^∗∗^ *p* < 0.01, ^∗∗∗^ *p* < 0.001, ^∗∗∗∗^ *p* < 0.0001; ns, not significant.

Given the pivotal role of gut oxidative stress, immune dysregulation, and barrier dysfunction in driving systemic autoimmunity, we investigated the local effects of LGG@PDA on intestinal ROS dynamics, mucosal immune cell populations, and epithelial barrier integrity. We first performed immunofluorescence staining of the intestinal tract. As shown in Figure 4A, there was a substantial reduction in ROS levels in the intestinal tract of LGG@PDA‐treated mice, confirming its robust ROS‐scavenging activity. Given the established role of oxidative stress in shaping the mucosal immune response, we next analyzed immune cell populations in the gut. Flow cytometric analysis of lamina propria lymphocytes showed that LGG@PDA treatment significantly reduced the proportion of Th17 cells among CD4^+^ T cells (Figure 4B), which was consistent with the immunomodulatory effects previously observed in the spleen.

As intestinal macrophages play a pivotal role in maintaining mucosal immune balance, orchestrating inflammatory responses, and facilitating tissue repair, we next examined macrophage subsets. LGG@PDA treatment significantly reduced the frequency of lamina propria macrophages, particularly those producing TNF‐α (Figure 4C,D). Subdivision based on CX3CR1 expression revealed a pronounced shift from CX3CR1^low/int^ pro‐inflammatory macrophages toward CX3CR1^hi^ tissue‐resident macrophages (Figure 4E,F), which are associated with tissue repair and apoptotic cell clearance. Consistent with these findings in the pristane‐induced model, analysis of lamina propria macrophages in *MRL/lpr* lupus‐prone mice showed comparable shifts in macrophage subsets following LGG@PDA treatment, supporting its effect on intestinal immune homeostasis (Figure S2).

Finally, the impact of LGG@PDA on intestinal barrier integrity was evaluated. Transcriptional profiling revealed upregulation of tight junction‐related genes, including *ZO‐1, Occludin, Claudin‐4*, and *Claudin‐23* (Figure 4G). Consistently, immunohistochemical analysis further confirmed increased protein expression of these molecules, indicating the restoration of intestinal epithelial barrier integrity following LGG@PDA treatment (Figure 4H). Collectively, these results suggest that LGG@PDA alleviates intestinal oxidative stress, rebalances mucosal immune responses, and reinforces barrier function, addressing critical pathogenic mechanisms underlying SLE.

### 2.4 LGG@PDA Reshapes Gut Microbiota Structure and Enhances Microbial Diversity in Lupus Mice

**FIGURE 5:**
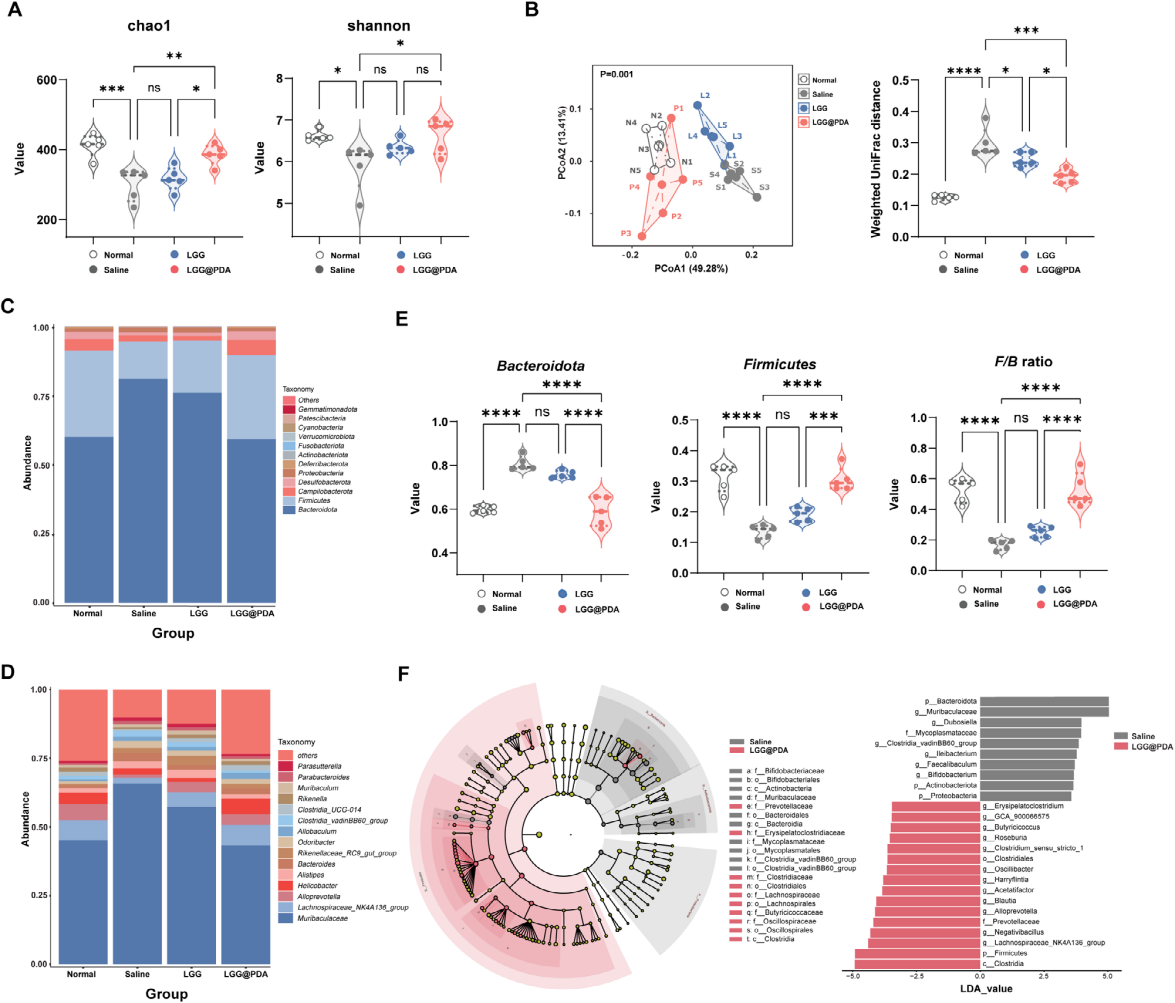
LGG@PDA reshapes gut microbiota composition and enhances microbial diversity in pristane‐induced lupus‐like mice. Gut microbiota was examined by 16S rRNA sequencing following treatments. (A) Alpha‐diversity of fecal samples assessed by Chao1 and Shannon indices. (B) Principal coordinate analysis (PCoA) based on weighted UniFrac distances. (C,D) Stacked bar plots of gut microbial composition at the phylum (C) and genus (D) levels. (E) Relative abundance of major phyla, including Firmicutes, Bacteroidetes, and the corresponding Firmicutes/Bacteroidetes ratio. (F) Linear discriminant analysis effect size (LEfSe) identifying differential taxa among groups, with a cladogram illustrating phylogenetic relationships. Key taxa enriched in Saline‐ and LGG@PDA‐treated mice (LDA > 2.5) are indicated. Each point represents one subject. Statistical significance was determined using one‐way ANOVA (A, B, D).  ^∗^ *p* < 0.05, ^∗∗^ *p* < 0.01, ^∗∗∗^ *p* < 0.001, ^∗∗∗∗^ *p* < 0.0001; ns, not significant.

Given that gut dysbiosis is increasingly recognized as a driver of autoimmunity, we next investigated whether LGG@PDA modulates gut microbial communities and their metabolic outputs in pristane‐induced lupus‐like mice. 16S rRNA gene sequencing revealed that lupus mice exhibited significant reductions in Chao1 and Shannon indices (Figure 5A), reflecting decreased microbial richness and diversity. Notably, LGG@PDA administration restored microbial diversity toward levels comparable to those of healthy controls, suggesting a corrective effect on lupus‐associated microbial loss. Analyses of the community structure further supported these findings. Principal coordinate analysis based on weighted UniFrac distances showed that the microbiota of LGG@PDA‐treated mice clustered closely with that of healthy controls, whereas those from saline‐treated and LGG‐treated mice remained distinctly separated (Figure 5B). In addition, heatmap visualization at both phylum and genus levels revealed a pronounced remodeling of gut microbial composition in lupus mice (Figure 5C,D). At the phylum level, LGG@PDA significantly increased the relative abundance of Firmicutes and concomitantly reduced Bacteroidetes, thereby elevating the Firmicutes/Bacteroidetes (F/B) ratio, a microbial signature previously associated with lupus severity (Figure 5E). Other phyla, such as Campylobacterota, Desulfobacterota, Proteobacteria, and Deferribacterota, showed no significant changes following LGG@PDA treatment. At the genus level, LGG@PDA administration reduced the relative abundance of *Muribaculaceae*, *Clostridia_vadinBB60_group*, and *Ileibacterium*, while enriched *Lachnospiraceae_NK4A136_group, Alloprevotella*, and *Roseburia*, which is generally considered beneficial for gut homeostasis.

To further identify key microbial taxa discriminating between groups, we performed linear discriminant analysis effect size (LEfSe) (Figure 5F). The cladogram illustrated distinct phylogenetic relationships among microbial communities between the control group and the LGG@PDA‐treated group. Saline‐treated mice were enriched in Bacteroidia class, Bacteroidales order and Muribaculaceae family, whereas LGG@PDA‐treated mice showed increased the relative abundance of Clostridia class, Oscillospirales order, and Lachnospiraceae family. Notably, *Lachnosporaceae_NK4A136_group* exhibited the highest LDA score in the LGG@PDA group, suggesting its pivotal role in mediating community restructuring (Figure 5F). These findings collectively indicate that LGG@PDA selectively reshapes gut microbial communities, restoring a more balanced composition consistent with improved host immune regulation.

### 2.5 LGG@PDA Reprograms Fecal Metabolites and Restores Efferocytosis

**FIGURE 6:**
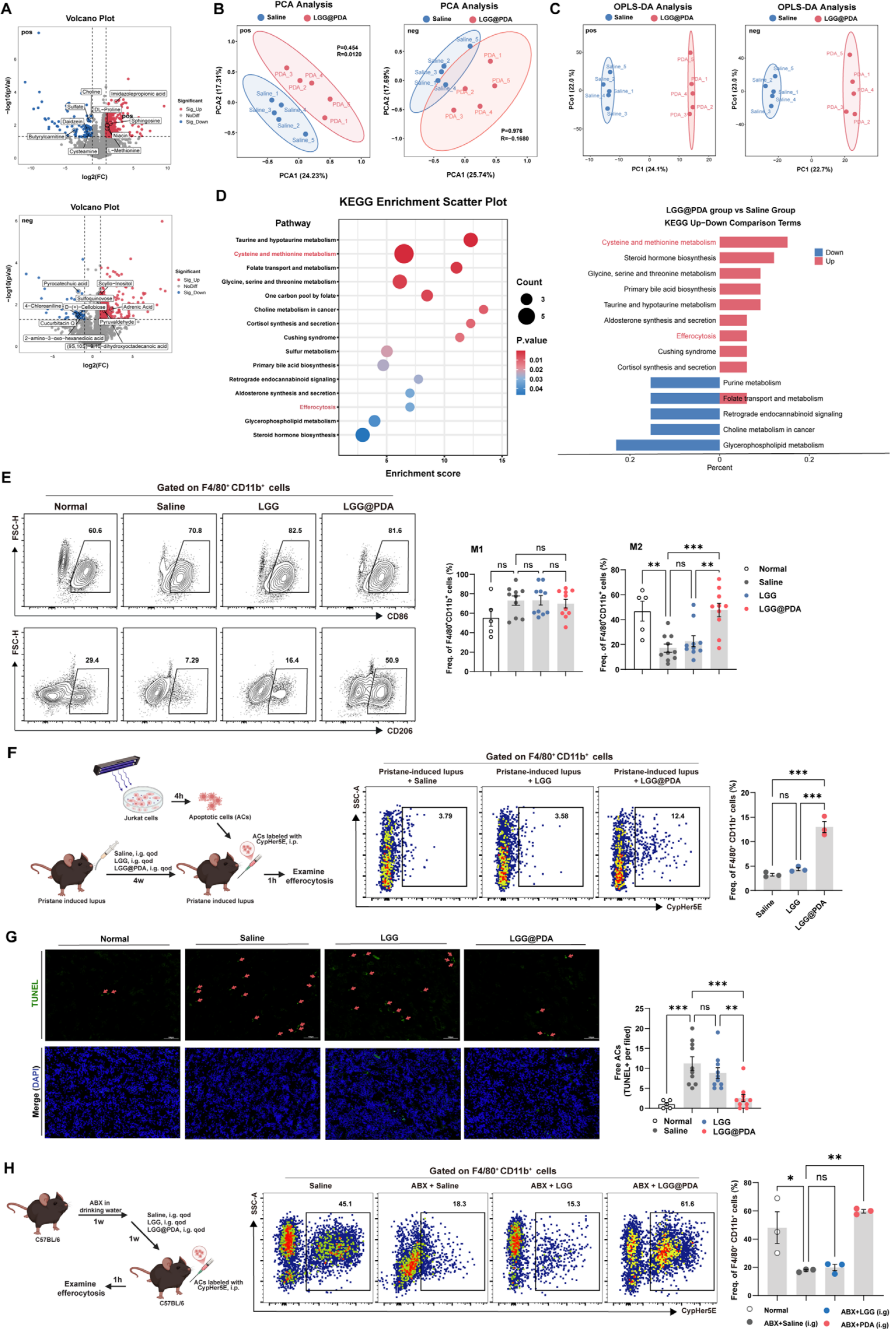
LGG@PDA reprograms fecal metabolites and restores macrophage efferocytosis in lupus mice. (A) Volcano plots of identified metabolites in positive and negative ion modes, with the five most abundant upregulated/downregulated metabolites highlighted (|log_2_FC| > 1, *p* < 0.05). (B) Principal component analysis (PCA) of fecal metabolite profiles in positive and negative ion modes. (C) Orthogonal partial least squares discriminant analysis (OPLS‐DA) score plots for positive and negative ion modes. (D) KEGG pathway enrichment analysis of differential metabolites (|log_2_FC| > 1, *p* < 0.05, VIP>1). (E) Flow cytometric analysis of peritoneal macrophage polarization (M1 and M2 macrophages were gated as follows: M1, Zombie Aqua^−^CD45^+^Ly6G^−^CD11b^+^F4/80^+^CD86^+^; M2, Zombie Aqua^−^CD45^+^Ly6G^−^CD11b^+^F4/80^+^CD206^+^). (F) Schematic illustration of efferocytosis assay in vivo (left) and quantification of peritoneal macrophage phagocytosis of CypHer5E‐labeled apoptotic Jurkat cells by flow cytometry (right, Zombie Aqua^−^CD45^+^CD11b^+^F4/80^+^CypHer5E^+^). (G) TUNEL staining of kidney sections for detection of uncleared apoptotic cells. (H) Experimental scheme for gut microbiota depletion using an antibiotic cocktail (ABX) in C57BL/6 mice and subsequent LGG@PDA supplementation followed by evaluation of macrophage efferocytosis capacity with flow cytometry (Zombie Aqua^−^CD45^+^CD11b^+^F4/80^+^CypHer5E^+^). Each point represents one individual subject, and bars indicate the mean ± SEM. Statistical significance was determined using one‐way ANOVA (E‐H).  ^∗^ *p* < 0.05, ^∗∗^ *p* < 0.01, ^∗∗∗^ *p* < 0.001, ^∗∗∗∗^ *p* < 0.0001; ns, not significant.

Given the central role of gut microbiota in shaping host metabolic outputs, non‐targeted metabolomic profiling of fecal samples was used to identify whether the LGG@PDA‐induced microbial remodeling was accompanied by alterations in fecal metabolite profiles. The results showed that the metabolites in fecal samples of pristane‐induced lupus‐like mice treated with LGG@PDA were distinct from those of the saline‐treated group. A total of 3634 and 2488 metabolites were identified in the positive and the negative ion modes, respectively (Figure 6A). Among these, 443 metabolites (355 upregulated, 88 downregulated) and 299 metabolites (242 upregulated, 57 downregulated) showed significant alterations (|log_2_FC| > 1, p < 0.05). In the positive ion mode, imidazolepropionic acid, DL‐proline, sphingosine, niacin, and L‐methionine ranked as the five most abundant upregulated metabolites, while choline, sulfate, daidzein, butyrylcarnitine, and cysteamine were identified as the most abundant downregulated metabolites. In the negative ion mode, the five most abundant upregulated metabolites included scyllo‐inositol, adrenic acid, pyruvaldehyde, sulfoquinovose, and (9S,10S)‐9,10‐dihydroxyoctadecanoic acid, whereas pyrocatechuic acid, 4‐chloroaniline, cucurbitacin, D‐(+)‐cellobiose, and 2‐amino‐3‐oxo‐hexanedioic acid were the predominant downregulated metabolites. Principal component analysis revealed distinct clustering between the two groups, suggesting significant metabolic reprogramming of fecal metabolite profiles. Notably, clusters were clearly separated in the positive ion mode, while partial overlap was observed in the negative ion mode (Figure 6B). To further discriminate between the two groups, orthogonal partial least squares discriminant analysis (OPLS‐DA) was performed. The OPLS‐DA score plots demonstrated distinct separation between LGG@PDA‐treated and saline‐treated groups in both positive and negative ion modes (Figure 6C). Model evaluation yielded *R* ^2^Y and *Q* ^2^ values of 0.998 and 0.808 in the positive ion mode, and 0.993 and 0.759 in the negative ion mode, suggesting adequate model fit and acceptable predictive reliability. These results indicated that LGG@PDA treatment led to a significant alteration in fecal metabolic profiles. Differential metabolites (|log_2_FC| > 1, p < 0.05, VIP > 1) were subsequently subjected to KEGG pathway enrichment analysis. The results revealed that upregulated metabolites in the LGG@PDA‐treated group were predominantly enriched in cysteine and methionine metabolism, as well as pathways associated with efferocytosis (Figure 6D).

Efferocytosis, the process by which phagocytes remove apoptotic cells, is fundamental for maintaining immune tolerance and tissue homeostasis. Defective efferocytosis is recognized as a critical feature of lupus pathogenesis. Based on the enrichment of the pathway related to efferocytosis in the metabolomic analysis, We evaluated the ability of LGG@PDA treatment to restore efferocytosis capacity in lupus mice. As shown in Figure 6E, LGG@PDA administration promoted macrophage polarization toward the M2 phenotype, which is associated with anti‐inflammatory activity, enhanced phagocytosis, tissue repair, and immunomodulation. To directly assess this, apoptosis was induced in Jurkat cells via UVB irradiation, and confirmed by flow cytometry (Figure S3). These apoptotic cells were subsequently labeled with CypHer5E and injected intraperitoneally into recipient mice. Compared with saline‐treated and LGG‐treated controls, macrophages isolated from LGG@PDA‐treated mice exhibited a significantly enhanced engulfment of CypHer5E‐labeled apoptotic Jurkat cells (Figure 6F). Consistently, histological examination revealed fewer uncleared apoptotic cells accumulated in the kidneys of LGG@PDA‐treated mice (Figure 6G), indicating that LGG@PDA may restore the efferocytosis function of lupus mice.

To further investigate whether the gut microbiota contributes to the restoration of efferocytosis, the intestinal microbes of C57BL/6 mice was depleted using broad‐spectrum antibiotics. Subsequently, the mice received oral gavage of either LGG or LGG@PDA for one week, followed by intraperitoneal injection of Cypher5E‐labeled apoptotic Jurkat cells. The results showed that the macrophage efferocytosis was markedly destroyed by antibiotic treatment, whereas LGG@PDA supplementation effectively restored this function (Figure 6H), indicating that gut‐derived signals may influence the systemic regulation of efferocytosis.

### 2.6 L‐methionine as a Metabolic Mediator Linking Gut Microbiota Remodeling to Efferocytosis and Lupus Amelioration

**FIGURE 7:**
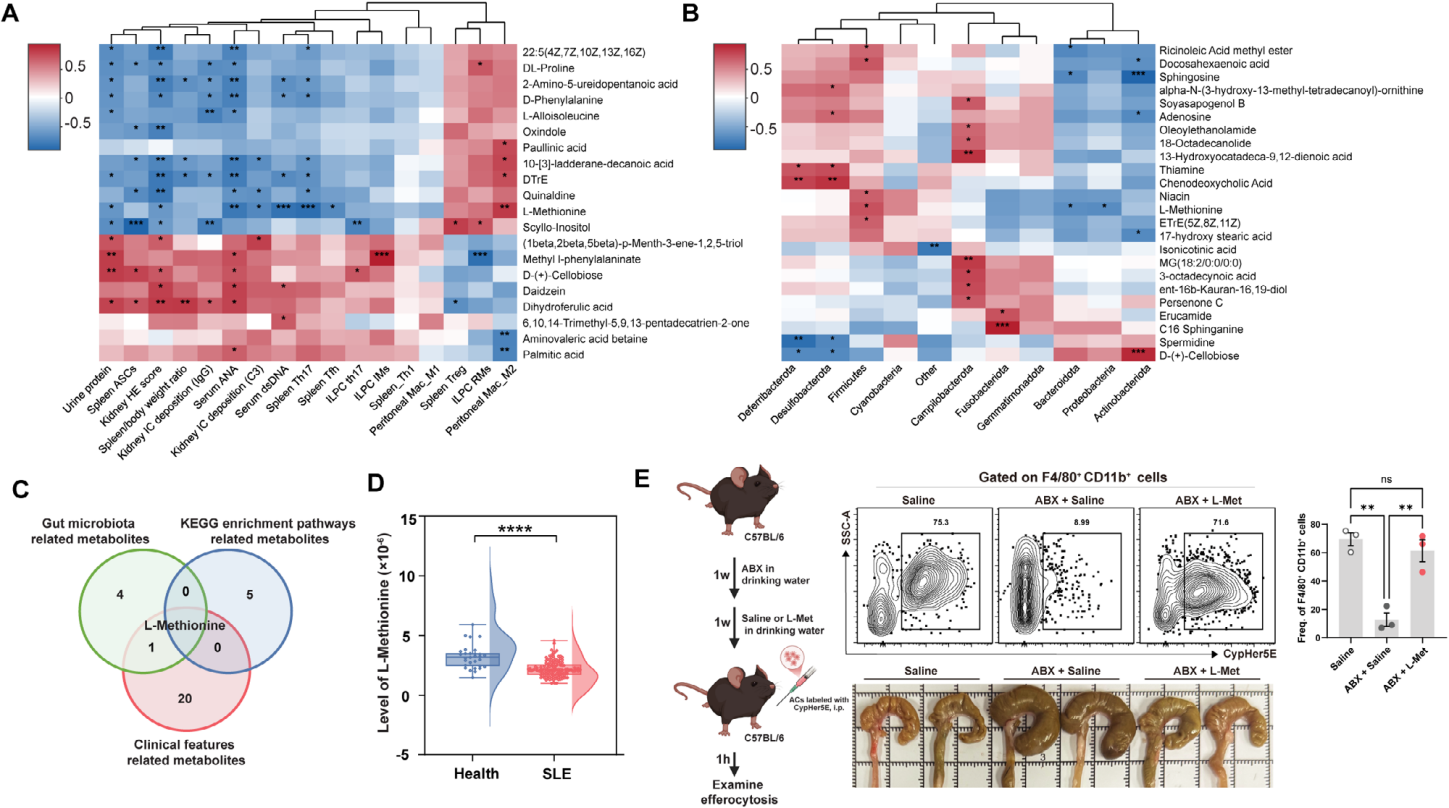
L‐methionine links gut microbiota remodeling to metabolic changes in lupus. (A) Correlation analysis between fecal metabolites and clinical features of lupus mice after LGG@PDA administration, displaying the 20 most abundant metabolites with significant correlations (*p* < 0.05). (B) Correlation analysis between fecal metabolites and gut microbiota abundance at the phylum level, showing the 20 most abundant metabolites among those with significant correlations (*p* < 0.05). (C). Venn diagram illustrating metabolites significantly associated with gut microbiota composition, clinical features and KEGG‐enriched pathways (efferocytosis and cysteine/methionine metabolism), with L‐methionine identified as an overlapping metabolite. (D). Serum levels of L‐methionine in patients with SLE (*n* = 133) and healthy controls (*n* = 30). (E) Experimental scheme for gut microbiota depletion using an antibiotic cocktail (ABX) in C57BL/6 mice followed by L‐methionine supplementation, with assessment of macrophage efferocytosis and cecal morphology (Zombie Aqua^−^CD45^+^CD11b^+^F4/80^+^CypHer5E^+^). Each point represents one individual subject, and bars indicate the mean ± SD. Statistical significance was determined using unpaired Student's test (D) and one‐way ANOVA (E). Pearson correlation was used for correlation analyses. ^∗^ *p* < 0.05, ^∗∗^ *p* < 0.01, ^∗∗∗^ *p* < 0.001, ^∗∗∗∗^ *p* < 0.0001; ns, not significant.

To further investigate the potential mechanisms underlying metabolite reprogramming and its impact on lupus pathogenesis, correlation analyses among lupus clinical phenotypes, gut microbiota composition, and fecal metabolites were performed. This analysis identified 22 metabolites significantly associated with disease severity (Table S1). Notably, L‐methionine, DTrE, D‐phenylalanine, and 2‐amino‐5‐ureidopentanoic acid exhibited negative correlations with key features of lupus, including proteinuria, anti‐dsDNA antibody, and ANA levels. Specifically, L‐methionine showed a strong inverse correlation with renal pathology and the frequencies of Th17 and Tfh cells in the spleen, while displaying a positive correlation with the frequency of peritoneal M2 macrophages (Figure 7A). In addition, six metabolites were identified to be positively correlated with the abundance of Firmicutes or negatively correlated with Bacteroidetes (Table S1). Notably, both ricinoleic acid methyl ester and L‐methionine were found to be linked to changes in these two phyla (Figure 7B). Through Venn diagram analysis, L‐methionine was identified as a central metabolite connecting cysteine/methionine metabolism, the efferocytosis pathway, gut microbiota and clinical features of lupus (Figure 7C). To assess its clinical relevance, serum levels of L‐methionine were measured in patients with SLE. As shown in Figure 7D, L‐methionine was significantly reduced compared with healthy controls, consistent with findings from lupus mice. Based on our previous observation that antibiotic treatment disrupts the gut microbiota and diminishes the capacity of macrophage efferocytosis, we next investigated whether the reduction of L‐methionine is functionally linked to this process. Mice receiving antibiotic treatment were then administered exogenous L‐methionine, followed by evaluation of peritoneal macrophage efferocytosis. The results showed that L‐methionine supplementation effectively restored efferocytosis impaired by antibiotic treatment and alleviated cecal enlargement (Figure 7E).

**FIGURE 8:**
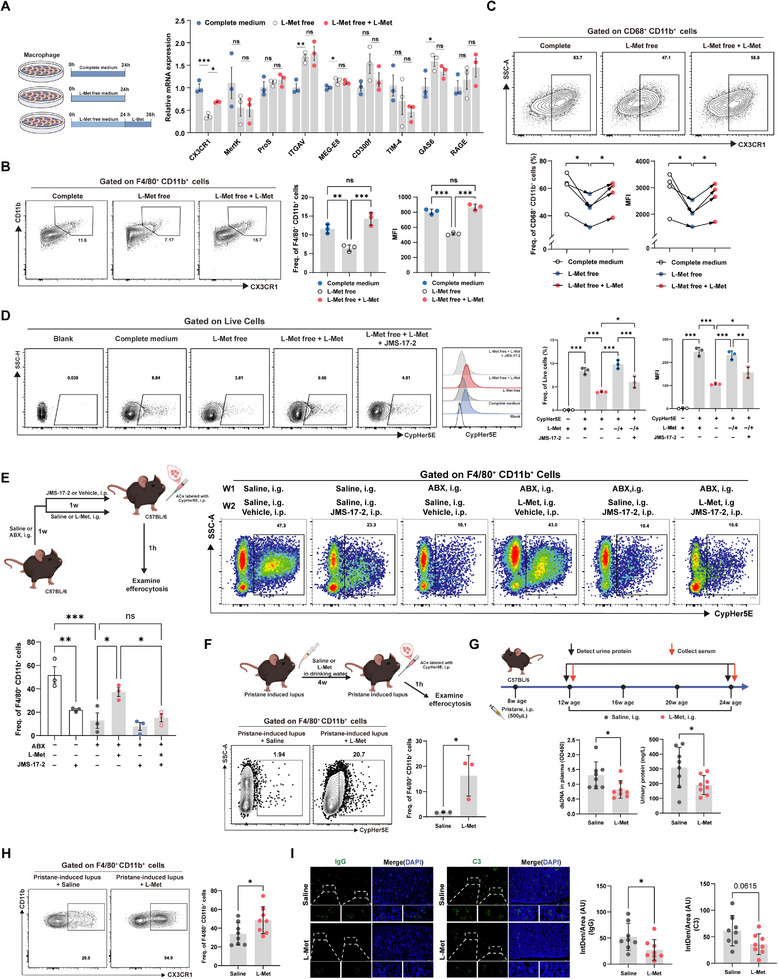
L‐methionine restores macrophage efferocytosis via CX3CR1 and ameliorates lupus manifestations. (A) Schematic illustration of L‐methionine deprivation and replenishment in RAW264.7 cells, followed by qPCR analysis of efferocytosis‐related genes. (B,C) Flow cytometric analysis of CX3CR1 levels in RAW264.7 cells (B, Zombie Aqua^−^CD11b^+^F4/80^+^) and human CD14^+^ monocyte‐derived macrophages (C, Zombie Aqua^−^CD45^+^CD11b^+^CD68^+^) after L‐methionine deprivation and replenishment, showing the frequency of CX3CR1^+^ macrophage and mean fluorescence intensity (MFI). (D) In vitro efferocytosis assay in RAW264.7 cells following L‐methionine deprivation, replenishment, and CX3CR1 blockade with JMS‐17‐2. Quantification includes the proportion of CypHer5E^+^ cells and MFI. (E) Experimental scheme for ABX‐mediated gut microbiota depletion in C57BL/6 mice with subsequent L‐methionine supplementation, and CX3CR1 blockade. Peritoneal macrophage efferocytosis was quantified by flow cytometry. (F) Schematic of exogenous L‐methionine supplementation in pristane‐induced lupus‐like mice, with subsequent quantification of peritoneal macrophage efferocytosis. (G) Experimental design for L‐methionine supplementation in pristane‐induced lupus‐like mice, with measurement of plasma anti‐dsDNA antibody and urinary protein levels. (H) Flow cytometric analysis of CX3CR1^+^ macrophages (Zombie Aqua^−^CD45^+^CD11b^+^F4/80^+^CX3CR1^+^) in peripheral blood following 12 weeks of L‐methionine supplementation. (I) Immunofluorescence staining and quantification of IgG (left) and C3 (right) deposition in renal glomeruli. Each point represents one individual subject, and bars indicate the mean ± SEM/SD. Statistical significance was determined using unpaired Student's *t*‐test (F–I) and one‐way ANOVA (A‐E). Pearson correlation was used for correlation analyses. ^∗^ *p* < 0.05, ^∗∗^ *p* < 0.01, ^∗∗∗^ *p* < 0.001, ^∗∗∗∗^ *p* < 0.0001; ns, not significant.

To further elucidate the molecular mechanism by which L‐methionine regulates efferocytosis, RAW264.7 macrophages were subjected to L‐methionine deprivation for 24 h followed by repletion for 12 h, and the expression of a panel of canonical efferocytosis genes was examined by quantitative PCR, including *CX3CR1, Mertk*, *ProS1*, *ITGAV*, *MFG‐E8*, *CD300f*, *TIM‐4*, *GAS6*, and *RAGE*. Among these genes, only CX3CR1 exhibited a consistent and reversible transcriptional response to L‐methionine manipulation, characterized by downregulated upon L‐methionine deprivation and restoration upon repletion (Figure 8A). Flow cytometric analysis further confirmed that L‐methionine deprivation significantly decreased CX3CR1 expression of macrophages, while supplementation restored its expression (Figure 8B). Notably, a similar upregulation of CX3CR1 was observed in intestinal macrophages from LGG@PDA‐treated lupus mice. To assess the translational relevance of this finding, human CD14^+^ monocytes isolated from peripheral blood were differentiated into macrophages and subjected to L‐methionine deprivation and repletion. Consistent with observations in RAW264.7 cells, CX3CR1 expression was significantly decreased under L‐methionine deprivation and restored upon repletion (Figure 8C), indicating that the regulation of CX3CR1 by L‐methionine is conserved in human macrophages.

Given the established role of CX3CR1 as a chemokine receptor that recognizes the “find‐me” signal CX3CL1 released by apoptotic cells and facilitates phagocyte migration toward apoptotic targets, we next examined whether CX3CR1 mediates the functional effects of L‐methionine on efferocytosis. Raw264.7 cells subjected to L‐methionine deprivation or repletion were co‐cultured with CypHer5E‐labeled apoptotic Jurkat cells. As shown in Figures 8D, L‐methionine deprivation resulted in a significant reduction in macrophage efferocytosis, whereas L‐methionine repletion restored the uptake of apoptotic cell. Notably, this restorative effect was abrogated by pharmacological inhibition of CX3CR1 using the selective antagonist JMS‐17‐2, indicating that CX3CR1 is required for the L‐methionine‐induced enhancement of macrophage efferocytosis. To further substantiate this mechanism in vivo, intraperitoneal administration of the JMS‐17‐2 in healthy mice impaired the efferocytic capacity of peritoneal macrophage, supporting a functional role for CX3CR1 in apoptotic cell clearance in vivo. Consistent with our previous observations, the restorative effect of L‐methionine on macrophage efferocytosis was abolished by CX3CR1 inhibition (Figure 8E).

Building on our findings that L‐methionine regulates macrophage efferocytosis in a CX3CR1‐dependent manner, we next examined its relevance to lupus disease outcomes. Pristane‐induced lupus‐like mice were administered exogenous L‐methionine, and the efferocytosis capacity of peritoneal macrophages was subsequently assessed. L‐methionine supplementation markedly enhanced macrophage efferocytosis compared with the controls (Figure 8F), corroborating the effects observed in LGG@PDA‐treated mice. Moreover, L‐methionine supplementation resulted in early reductions in anti‐dsDNA antibody levels and urinary protein excretion (Figure 8G), accompanied by increased CX3CR1 expression on peripheral macrophages (Figure 8H). With sustained treatment, these immunological improvements were followed by a marked attenuation of renal immune complex deposition and renal pathology (Figure 8I). Together, these findings support a role for microbiota‐derived L‐methionine in restoring macrophage efferocytosis through CX3CR1 regulation and conferring protection against lupus progression.

## 3 Discussion

SLE is a chronic autoimmune disease characterized by multisystem involvement and immune dysregulation, with increasing evidence implicating gut microbiota alteration in disease progression. [1, 6, 7, 37]. Probiotics interventions have shown potential in modulating immune responses and alleviating lupus‐like manifestations [15, 16, 18, 38]. Nevertheless, the efficacy of conventional probiotics is constrained by poor stability, rapid clearance, and limited colonization under inflammatory and oxidative conditions, highlighting the need for improved delivery strategies [39]. In this study, polydopamine‐encapsulated LGG (LGG@PDA) was developed to enhance its persistence and evaluated its therapeutic effects in both pristane‐induced and spontaneous lupus‐prone mice. PDA encapsulation not only improved bacterial survival and adhesion but also conferred antioxidative properties by scavenging reactive oxygen species, thereby fostering a favorable intestinal microenvironment for microbial restoration [18, 40, 41].

Consistent with previous reports demonstrating the immunomodulatory potential of probiotic supplementation [18], administration of unmodified LGG produced limited improvements in lupus‐like manifestations. In contrast, LGG@PDA treatment displayed more pronounced amelioration of clinical features, including reductions in proteinuria, serum autoantibody levels, and renal pathology. These therapeutic effects were accompanied by systemic immune modulation, characterized by the restoration of Th17/Treg balance and suppression of Tfh cell and ASC differentiation in the spleen, effectively counteracting the immune dysregulation observed in SLE [42].

Beyond its systemic effects, LGG@PDA exerted distinct immunomodulatory actions in the gut. It reduced intestinal oxidative stress, reestablished the Th17/Treg balance and reshaped macrophage populations by decreasing TNF‐α–producing pro‐inflammatory macrophages and expanding CX3CR1^hi^ tissue‐resident macrophages, leading to a shift toward a reparative immune environment [43, 44, 45]. Intestinal barrier disruption has been reported to facilitate the translocation of pathobionts, a process that may contribute to lupus pathogenesis [32, 33, 34]. Compared with LGG alone, LGG@PDA substantially restored epithelial barrier integrity by increasing expression of tight junction proteins. In addition, LGG@PDA markedly reshaped gut microbial composition and reprogrammed host metabolic outputs, including an increase in the Firmicutes/Bacteroidetes (F/B) ratio, which has been linked to disease severity in SLE [32, 33]. Collectively, these findings indicate that LGG@PDA enhances probiotic persistence and coordinates systemic and mucosal immunomodulation, offering a promising strategy for SLE management.

Of particular interest, fecal metabolomic profiling revealed an enrichment of metabolites associated with the efferocytosis pathway, a critical macrophage‐mediated process for apoptotic cell clearance and maintenance of immune tolerance. Impaired efferocytosis is increasingly recognized as a hallmark of lupus pathogenesis, leading to persistent autoantigen exposure and chronic inflammation [2, 46]. We found that LGG@PDA restored efferocytic capacity in peritoneal macrophages of lupus mice. Moreover, depletion of the gut microbiota by antibiotics impaired macrophage efferocytosis, which could be rescued by LGG@PDA treatment, highlighting the contribution of microbiota‐derived signals in maintaining the function of efferocytosis [47]. Metabolomic profiling further revealed that LGG@PDA administration increased the abundance of L‐methionine, a metabolite correlated with lupus severity and gut microbiota alterations in treated mice. Moreover, its levels were reduced in SLE patients compared with healthy controls. Functionally, L‐methionine supplementation reversed the defective efferocytosis observed in lupus mice and rescued the impairment of peritoneal macrophage efferocytosis induced by antibiotic‐mediated gut microbiota depletion. Mechanistically, L‐methionine deprivation markedly suppressed CX3CR1 expression in both RAW264.7 and human CD14^+^ monocyte‐derived macrophages, whereas replenishment restored its expression. Consistent with this regulation, blockade of CX3CR1 abolished the ability of L‐methionine to maintain macrophage efferocytosis in both in vitro and in vivo. CX3CR1 functions as a chemokine receptor that recognizes the “find‐me” signal CX3CL1 released by apoptotic cells, thereby guiding phagocyte migration and promoting efficient efferocytosis [48, 49, 50]. Previous studies have demonstrated that loss of CX3CR1 impairs apoptotic cell removal and promotes inflammation, underscoring its pivotal role in maintaining immune tolerance [50, 51, 52]. Additionally, in lupus‐prone models such as *MRL/lpr* mice exacerbates disease manifestations, including glomerulonephritis [53]. Extending these observations, we found that microbiota‐derived L‐methionine regulates CX3CR1 expression in macrophages, which in turn supports efferocytosis. Consistent with this mechanism, exogenous L‐methionine supplementation in pristane‐induced lupus‐like mice enhanced peritoneal macrophage efferocytosis, increased CX3CR1 expression in circulating macrophages, attenuated the elevation of anti‐dsDNA antibodies and proteinuria, and mitigated renal immune complex deposition. Collectively, these findings delineate a mechanistic axis linking gut microbiota–derived L‐methionine to CX3CR1‐dependent macrophage efferocytosis, providing a functional bridge between intestinal metabolic cues and systemic immune regulation in lupus.

Nevertheless, several limitations should be acknowledged. First, although LGG@PDA demonstrated therapeutic efficacy in murine models of lupus, its translation to human SLE requires validation in larger clinical cohorts. In addition, the long‐term safety and stability of LGG@PDA were not evaluated, which are essential for future clinical application. Notably, methionine metabolism is tightly linked to one‐carbon metabolism and SAM‐dependent epigenetic regulation, which may contribute to L‐methionine–mediated modulation of CX3CR1 expression. While gut microbiota modulation has been reported to partially restore aberrant DNA methylation in SLE [54, 55], the long‐term epigenetic and immunological effects of sustained L‐methionine supplementation require careful evaluation in future studies. Addressing these issues in subsequent studies will be critical to establish the feasibility, safety, and robustness of this probiotic‐based therapeutic strategy.

## 4 Conclusions

In summary, this study established a bioengineered LGG@PDA with enhanced bacterial viability, adhesion, and antioxidative capacity for the treatment of SLE. In murine lupus models, LGG@PDA alleviated disease manifestations by reducing autoantibody levels, improving renal pathology, restoring gut and immune homeostasis, and enhancing macrophage efferocytosis. Mechanistically, these effects were associated with microbiota‐derived L‐methionine, which promoted macrophage efferocytosis through the upregulation of CX3CR1 expression, bridging gut microbial metabolism with systemic immune regulation. Collectively, these findings highlight LGG@PDA as a promising probiotic‐based intervention for SLE and identify L‐methionine‐mediated regulation of macrophage efferocytosis as a potential mechanistic axis underlying its therapeutic benefit.

## 5 Experimental Section

### 5.1 Preparation and Characterization of LGG@PDA

#### 5.1.1 Bacterial Culture and Stock

*Lactobacillus rhamnosus GG* (ATCC 53103, #01090P) was purchased from the China Center of Industrial Culture Collection (CICC). The initially revived colonies were further identified by matrix‐assisted laser desorption/ionization time‐of‐flight mass spectrometry (MALDI‐TOF MS). Routine cultivation was performed in de Man, Rogosa and Sharpe (MRS) agar medium or broth at 37°C under anaerobic conditions. For long‐term storage, bacterial suspensions were mixed with 15% (v/v) glycerol, dispensed into anaerobic glass vials, and preserved at −80°C until subsequent use.

#### 5.1.2 Preparation of LGG@PDA

LGG@PDA was synthesized by incubating LGG with dopamine hydrochloride in 0.01 M Tris‐HCl buffer (pH 8.8) under constant rotation. 5 × 10^9^ colony forming units (CFU) of LGG were dispersed in 10 mL of Tris‐HCl containing different concentrations of dopamine hydrochloride (0.5, 1.0, and 2.0 mg mL^−1^). The resulting mixture was shaken for 30 min at room temperature. After washing with phosphate buffered saline (PBS) for three times, LGG@PDA was obtained by centrifugation at 1500*×g*.

#### 5.1.3 Characterization of LGG and LGG@PDA

The morphological features of LGG and LGG@PDA were examined using transmission electron microscopy (TEM, Talos L120C G2). For sample preparation, 10 µl of bacterial suspension was dropped onto a 300‐mesh copper grid pre‐coated with formvar/carbon, followed by negative staining with phosphotungstic acid. The hydrodynamic diameters and zeta potentials of LGG, LGG@PDA (0.5, 1.0 and 2.0 mg mL^−1^) were analyzed using a Zetasizer Nano (Malvern, UK). All measurements were conducted in triplicate under standardized conditions to ensure reproducibility.

### 5.2 In Vitro Tolerance and Antioxidant Assays

#### 5.2.1 In Vitro Tolerance Assay

The tolerance of LGG@PDA to simulate gastrointestinal conditions was assessed by exposing bacterial suspensions to artificial gastric juice (pH 2.0, containing 1% (w/v) pepsin) and then incubated in intestinal fluid (pH 7.4, containing 0.5% bile salts and 1% Trypsin) for 4 h at 37°C. Bacterial viability was quantified at predetermined time points by plating serial dilutions on MRS agar.

#### 5.2.2 In Vitro Antioxidant Assay

Bone marrow–derived macrophages (BMDMs) were obtained from the femurs and tibias of C57BL/6 mice. Briefly, bone marrow cells were collected by flushing with DMEM medium supplemented with 10% fetal bovine serum (FBS), followed by filtration through a 70 µm cell strainer to generate a single‐cell suspension. Red blood cells were eliminated using ammonium‐chloride‐potassium (ACK) lysis buffer for 3–5 min at room temperature and subsequently washed with DMEM. The remaining cells were maintained in complete DMEM containing 10% FBS, 1% penicillin/streptomycin, and 20 ng mL^−1^ macrophage colony‐stimulating factor (M‐CSF, MedChemExpress, #HY‐P7085) at 37°C under a humidified 5% CO_2_ atmosphere. Culture medium was refreshed every 2–3 days, and adherent cells differentiated into macrophages after approximately 7 days.

For antioxidant activity, BMDMs were seeded into 12‐well plates and co‐cultured with either LGG or LGG@PDA at a bacterial‐to‐ratio of 5:1 for 24 h, followed by stimulation with lipopolysaccharide (LPS, 100 ng mL^−1^) for 4 h. Intracellular reactive oxygen species (ROS) levels were then detected using a DCFH‐DA probe (Solarbio, #CA1410) according to the manufacturer's instructions. Cell nuclei were stained with Hoechst 33342 (Beyotime, #C1028) for visualization, and fluorescence images were acquired using a fluorescence microscope (Nikon ECLIPSE Ti2‐U). Quantification of ROS fluorescence intensity was performed using ImagaJ software.

### 5.3 Growth Curve and Cell Counting Kit‐8 (CCK‐8) assay

The growth profiles of LGG and LGG@PDA were assessed in MRS broth at 37°C under anaerobic conditions. The optical density at 600 nm (OD_600_) was recorded every 2 h. For bacterial viability assessment, cultures at the logarithmic phase were subjected to the CCK‐8 assay, and absorbance was measured to evaluate bacterial metabolic activity.

### 5.4 In Vivo Tracking of Gastrointestinal Retention

#### 5.4.1 Cy5 Labeled LGG

LGG was fluorescently labeled with Cy5‐NHS ester (MedChemExpress, #HY‐D0819) according to a modified chemical conjugation protocol [56, 57]. Briefly, bacterial suspensions containing approximately 10^8^ CFU mL^−1^ were incubated with 2 µM Cy5‐SE in PBS for 30 min at room temperature with gentle agitation. Following incubation, the bacteria were collected by centrifugation and washed three times with PBS to remove unreacted dye. The purified Cy5‐labeled LGG (LGG‐Cy5) was resuspended in PBS for subsequent experiments. Successful conjugation was further confirmed by flow cytometry, which detected the Cy5 fluorescence signal associated with LGG.

#### 5.4.2 Tracking of Gastrointestinal Retention

To evaluate gastrointestinal retention, equal doses of LGG‐Cy5 and LGG@PDA‐Cy5 were administered to mice by oral gavage. At 4, 8, and 12 h post‐administration, the gastrointestinal tract was excised for fluorescence imaging using an IVIS Spectrum system to monitor the distribution and clearance of Cy5 labeled LGG in the gastrointestinal tract.

To further evaluate colonization and proliferation of LGG, intestinal contents from different segments of gastrointestinal tract were collected, and total bacterial DNA was extracted using the fecal genome DNA extraction kit (TIANGEN; #DP328‐02) at 24 and 48 h post‐administration. Quantification of LGG was performed by quantitative PCR (q‐PCR) with LGG‐specific primers, which target the 16S rRNA gene. qPCR reactions were carried out in a 10 µL system containing 5 µL of ChamQ Universal SYBR qPCR Master Mix (Vazyme; #Q711‐02), 0.2 µM of each primer, and 10 ng of template DNA. Amplification was performed under the following conditions: 95°C for 5 min, followed by 40 cycles of 95°C for 15 s and 60°C for 30 s. Melting curve analysis was conducted to confirm specificity. Relative bacterial abundance was calculated using the formula 2^−ΔΔCt^ (where ΔΔCt = ΔCt of experimental group—ΔCt of control group) with total bacterial 16S rRNA as the internal reference. The detailed primer sequences for LGG and 16S rRNA were listed in Table S2.

### 5.5 Animal Experiments

All animal experiments were conducted following the institutional guidelines for the care and use of laboratory animals at the institute of Dermatology, Chinese Academy of Medical Sciences, with approval from the local regulatory authorities (approval number 2022‐DW‐017 and 2025‐DW‐004). Female C57BL/6J mice and *MRL/lpr* were purchased from SPF (Beijing) Biotechnology Co., Ltd. and housed under specific pathogen‐free (SPF) conditions with free access to standard laboratory chow and water. Animals were housed on wood‐chip bedding in a temperature‐controlled environment under a 12 h light/dark cycle. At the beginning of the study, all mice were healthy, drug‐naïve, and had not been subjected to any prior experimental manipulation. To reduce inter‐individual variation in gut microbiota composition, mice were co‐housed for two weeks before interventions to equilibrate microbial communities across cages and litters. For pristane‐induced lupus‐like murine model, eight‐week‐old female C57BL/6J mice received an intraperitoneal injections of 500 µL pristane (Millipore Sigma, P9622).

#### 5.5.1 LGG and LGG@PDA Oral Experiments

One month after lupus induction with pristane, mice were assessed for urinary protein, anti‐dsDNA antibodies, and ANA levels. After randomization to minimize intergroup differences, oral administration was initiated. Pristane‐induced lupus‐like mice were treated via intragastric gavage with a total dose of 5 × 10^8^ CFU of LGG or LGG@PDA, or saline (100 µL dose^−1^), three times per week. Age‐matched healthy mice without pristane induction were included as a normal control group and received saline by intragastric administration following the same schedule. In parallel, *MRL/lpr* mice were divided into three groups and treated with saline, LGG, or LGG@PDA using the same dosing regimen and administration schedule. Urinary protein levels, anti‐dsDNA antibodies, and ANA titers were assessed throughout the treatment period. At the study endpoint, mice were euthanized, and tissues including spleen, draining lymph nodes, intestinal tract, and kidneys were collected for subsequent analysis.

#### 5.5.2 Antibiotic Treatment

Mice were pretreated with antibiotic in drinking water for at least 7 days before experiments. The antibiotic cocktail consisted of 1 g L^−1^ ampicillin (Macklin, #A800429), 1 g L^−1^ neomycin sulfate (Macklin, #N814740), 1 g L^−1^ metronidazole (Macklin, #M813526), 0.5 g L^−1^ vancomycin (Macklin, #V871983).

#### 5.5.3 L‐Methionine Treatment

Mice assigned to methionine treatment were provided with drinking water supplemented with 50 mM L‐methionine (MedChemExpress, #HY‐N0326).

#### 5.5.4 CX3CR1 Antagonist Treatment

Mice assigned to CX3CR1 antagonist treatment were administered JMS‐17‐2 (MedChemExpress, # HY‐123918) via intraperitoneal injection at a dose of 10 mg kg^−1^ every other day.

### 5.6 Assessment of Anti‐dsDNA and Anti‐Nuclear IgG Antibodies Levels by ELISA

Serum was obtained from mice at indicated time points throughout the treatment period via orbital venous plexus sampling under anesthesia. Levels of anti‐dsDNA and anti‐nuclear IgG antibodies were quantified using ELISA kits (CUSABIO, #CSB‐E11194m and CSB‐E12912m, respectively), following the manufacturer's instructions. For detection, serum was diluted 1:10 for anti‐dsDNA antibody and 1:100 for ANA measurements.

### 5.7 Assessment of Renal Injury

Urine samples were aseptically collected from mice throughout the treatment period. Proteinuria levels were quantitatively assessed using a Coomassie Brilliant Blue protein assay kit (Nanjing Jiancheng Bioengineering Institute, #C035‐2‐1). Renal histopathology was evaluated by paraffin embedding of the kidneys fixed in 4% paraformaldehyde (PFA), followed by preparation of 5‐µm sections. Hematoxylin and eosin (H&E) and periodic acid‐Schiff (PAS) staining were performed according to standard protocols to assess tissue morphology and pathological damage. The severity of glomerulonephritis was evaluated as the average score across 20 glomeruli. The grading criteria were defined as follows [58, 59]: 0, normal; 1, cellular proliferation or infiltration; 2, mesangial proliferation, hyaline deposition, or lobular formation; 3, global sclerosis or crescent formation.

Immunofluorescence analysis of IgG and C3 deposition was performed on 5‐µm OCT‐embedded frozen kidney sections. IgG deposits were identified using a goat anti‐mouse IgG2a heavy‐chain antibody conjugated with FITC (Abcam, #ab97244, 1:500). For C3 deposits detection, sections were incubated with a C3/C3b/C3c polyclonal antibody (Proteintech, #21337‐1‐AP, 1:500), followed by a FITC‐conjugated goat anti‐rabbit IgG antibody (Proteintech, #SA00003‐2, 1:200). Fluorescence images were collected with a fluorescence microscope (Olympus, BX53F2) Mean fluorescence intensities of IgG‐FITC and C3‐FITC were quantified using ImageJ software.

### 5.8 Assessment of Intestinal Barrier and ROS Generation

#### 5.8.1 Assessment of Intestinal Barrier

Total RNA was extracted from colonic tissues using Total RNA Extraction Reagent (Vazyme, #R401‐01‐AA). RNA purity and concentration were determined with a NanoDrop spectrophotometer (Thermo Scientific, ND‐2000). Reverse transcription was performed using the HiScript IV All‐in‐One Ultra RT SuperMix for qPCR (Vazyme, #R433) in accordance with the manufacturer's instructions. qRT‐PCR was carried out on a Roche LightCycler 480 II system with SYBR Premix Ex Taq II (Vazyme, #Q711‐02). *β‐actin/GAPDH* served as internal control, and relative gene expression was determined using the 2^−ΔΔCt^ method. The primer sequences for *ZO‐1, Occludin, Claudin‐1, Claudin‐2, Claudin‐4, Claudin‐23, β‐actin* and *GAPDH* are listed in Table S2.

For protein‐level analysis, colonic tissues were fixed in 4% PFA, dehydrated, embedded in paraffin, and sectioned at a thickness of 5 µm. After deparaffinization and rehydration, antigen retrieval was performed in citrate buffer (pH 6.0) using a microwave oven for 15 min. Endogenous peroxidase activity was quenched by incubation with 3% hydrogen peroxide for 10 min at room temperature, followed by blocking with 5% bovine serum albumin (BSA) for 30 min. Sections were incubated overnight at 4°C with primary antibodies against ZO‐1 (Proteintech, #21773‐1‐AP, 1:2000), Occludin (Proteintech, #27260‐1‐AP, 1:5000), Claudin‐4 (Proteintech, #16195‐1‐AP, 1:200), and Claudin‐23 (Invitrogen, #PA5‐103748, 1:200). After washing, sections were treated with HRP‐conjugated secondary antibodies for 1 h at room temperature and visualized using a DAB substrate kit. Hematoxylin was used for counterstaining. Images were captured with a light microscope, and staining intensity was quantified using ImageJ software.

#### 5.8.2 Assessment of ROS Generation

Colonic tissues were embedded in optimal cutting temperature (OCT) compound, snap‐frozen in liquid nitrogen, and cryosectioned at 5 µm thickness. Sections were equilibrated at room temperature for 10 min and rinsed with PBS to remove residual OCT. ROS production in colonic tissues was examined using OCT‐embedded frozen sections incubated with 10 µM dihydroethidium (DHE, Medchemexpress, #HY‐D0079) fluorescent probe for 30 min at 37°C. Fluorescence signals were captured with a fluorescence microscope (Olympus, BX53F2) and quantified using ImageJ software.

### 5.9 Isolation of Intestinal Immune Cells From Intestines Lamina Propria

Small intestine and colon were dissected, opened longitudinally, and washed with ice‐cold PBS to remove luminal contents. Adipose tissue and Peyer's patches were removed. Epithelial cells were removed by sequential incubation in PBS containing 1 mM DTT and 10 mM HEPES, followed by PBS with 30 mM EDTA and 10 mM HEPES, each step performed with shaking at 250 rpm for 10 min. The remaining tissue was minced (<0.5 cm) and digested in complete RPMI‐1640 medium containing Collagenase VIII (Sigma, # C2139, 100 U mL^−1^ for small intestine and 400 U mL^−1^ for colon) and DNase I (0.15 mg mL^−1^) at 37°C for 90 min. The digested suspension was filtered, centrifuged, and mononuclear cells were enriched using Percoll gradients (Cytiva, #17089101) of varying densities. Cells were washed, resuspended in complete RPMI‐1640 medium, and viability was assessed using trypan blue exclusion. Finally, cells were counted and adjusted to the desired concentration for downstream flow cytometry analysis

### 5.10 Flow Cytometry

Single‐cell suspensions were prepared from mouse spleen tissues (mechanically dissociated and filtered), intestines lamina propria and peritoneal lavage fluid. Approximately 1.5 × 10^6^ cells were treated with mouse Fc receptor blocking reagent (BD Pharmingen, #553141) for 15 min at room temperature, followed by surface staining using specific marker antibodies for 45 min at 4°C in the dark. For intracellular staining, cells were fixed and permeabilized using a Transcription Factor Buffer Set (BD Pharmingen, #562574), followed by staining with fluorescent antibodies for 1.5 h at 4°C. To assess cytokine production, cells were stimulated with Leukocyte Activation Cocktail (BD GolgiPlug antibody, BD,#550583) for 6 h at 37°C. Flow cytometric acquisition was performed using a Cytek NL‐CLC V16B14R8 or a BD LSRFortessa Cell Analyzer. Data analysis was conducted with FlowJo software. The specific antibodies used for surface and intracellular staining were listed in Table S3.

### 5.11 16S rRNA Sequencing and Bioinformatic Analysis

Stool samples were snap‐frozen immediately after collection and stored at −80°C until use. Bacterial genomic DNA was extracted, and the V3–V4 hypervariable region of the bacterial 16S rRNA gene was amplified. Amplicons were pooled and sequenced on an Illumina NovaSeq 6000 platform (Illumina, San Diego, CA) with paired‐end 250 bp reads (OE Biotech Co., Ltd., Shanghai, China). Raw sequences were processed with QIIME2 (v2020.11), including quality filtering, denoising, chimera removal, and taxonomic assignment against the SILVA 138 database. Alpha diversity indices (Chao1, Shannon, Observed_species, and Simpson) were calculated to estimate microbial richness and diversity. Beta diversity was assessed by weighted UniFrac distances, which were used for principal coordinates analysis (PCoA) and phylogenetic tree construction. Detailed experimental procedures are provided in the Supporting Methods.

### 5.12 Untargeted Metabolomics Analysis by LC–MS/MS

#### 5.12.1 5LC‐MS/MS Analysis of Stool Samples From Pristane‐Induced Lupus‐Like Mice

Stool samples from pristane‐induced lupus‐like mice were collected, snap‐frozen, and stored at −80°C until metabolomic analysis. Raw data were processed with XCMS v4.5.1, and metabolite annotation was performed based on accurate mass, retention time, MS/MS fragmentation, and isotopic distribution. Principal Component Analysis (PCA) and Orthogonal Partial Least‐Squares‐Discriminant Analysis (OPLS‐DA) were applied to assess sample distribution and identify differential metabolites. Variable Importance of Projection (VIP) values, along with *p*‐values from two‐tailed Student's *t*‐tests and fold change (FC), were used to define significant metabolites (VIP > 1.0, *p* < 0.05, |log_2_FC| > 1). Selected metabolites were further analyzed for KEGG pathway enrichment. Detailed LC‐MS/MS acquisition and data processing parameters were provided in the Supporting Methods.

#### 5.12.2 LC‐MS/MS Analysis of Serum From SLE Patients and Healthy Controls

The dataset analyzed in this study was obtained from the work of Zhang WQ, et al., 2022 [60], with sample collection, processing, and data generation performed exactly as described in the original publication.

### 5.13 Assessment of Macrophage Efferocytosis In Vitro and In Vivo

Apoptosis was induced in human Jurkat T cells (ATCC, #TIB‐152) cultured in RPMI‐1640 medium supplemented with 5% fetal bovine serum by exposure to ultraviolet B irradiation at a dose of 360 mJ. Following irradiation, cells were incubated at 37°C with 5% CO_2_ for 4 h to promote apoptosis, which was subsequently verified using an Annexin V‐FITC Apoptosis Detection Kit (Yeasen, #40302ES50). Apoptotic cells were then labeled with the pH‐sensitive dye CypHer5E NHS Ester (Cytiva, #PA15401) according to the manufacturer's instructions and resuspended in PBS prior to use in the efferocytosis assay.

#### 5.13.1 In Vivo Efferocytosis

A total of 1 × 10^6^ CypHer5E‐labeled apoptotic Jurkat cells were administered intraperitoneally into mice, which were euthanized after 1 h. Peritoneal fluid was collected by lavage using 8 mL of RPMI‐1640 medium. Efferocytosis was quantified by flow cytometry as the proportion of CypHer5E^+^ cells within the CD11b^+^ F4/80^hi^ peritoneal macrophage population.

#### 5.13.2 In Vitro Efferocytosis

CypHer5E‐labeled apoptotic Jurkat cells were co‐cultured with macrophages at a 1:1 ratio for 1 h. After incubation, macrophages were collected, stained with marker antibodies, and efferocytosis was quantified by flow cytometry as the proportion of CypHer5E^+^ cells within the macrophage population. The specific antibodies used for surface staining were listed in Table S3.

### 5.14 Isolation and Differentiation of Human CD14^+^ Monocyte‐Derived Macrophages

All human subjects in this study were approved by the ethics committee of the Institute of Dermatology, Chinese Academy of Medical Sciences (2024‐KY‐004) and all participants provided written informed consent. Human peripheral blood mononuclear cells (PBMCs) were isolated from healthy donor blood using Ficoll‐Paque density gradient centrifugation. CD14^+^ monocytes were then purified from PBMCs using MojoSort Human CD14 Selection Kit according to the manufacturer's instructions (Biolegend, #480025). Purified monocytes were cultured in RPMI‐1640 medium supplemented with 10% fetal bovine serum and 50 ng mL^−1^ recombinant human M‐CSF (MedChemExpress, # HY‐P7050) at 37°C with 5% CO_2_ for 7 days to induce differentiation into macrophages. The medium was refreshed every 2–3 days. Differentiation was confirmed by morphological assessment and surface marker expression (CD11b^+^, CD68^+^) using flow cytometry. The specific antibodies used for surface staining were listed in Table S3.

### 5.15 L‐Methionine Deprivation and Replenishment In Vitro

RAW264.7 cells (ATCC, #TIB‐71) and human CD14^+^ monocyte‐derived macrophages were subjected to L‐methionine deprivation and replenishment to evaluate the effects of methionine availability on macrophage function. Briefly, 5 × 10^5^ cells were cultured in methionine‐free DMEM for 24 h to induce methionine deprivation. For replenishment, the medium was replaced with complete DMEM medium, and cells were incubated for an additional 12 h. Following treatment, cells were collected for qPCR and flow cytometric analyses. The primer sequences were listed in Table S2, and the specific antibodies used for surface staining were summarized in Table S3.

### 5.16 Statistical Analysis

All statistical analyses were performed using IBM SPSS Statistics version 24.0 (SPSS Inc., Chicago, IL, USA). Continuous variables were presented as mean ± standard deviation/standard error of the mean (SD/SEM), while categorical variables were summarized as counts with percentages. Between‐group comparisons were conducted using the unpaired Student's *t* test or one‐way ANOVA for normally distributed data, and the Mann–Whitney *U* test for non‐normally distributed data. Categorical variables were analyzed using either the chi‐square test or Fisher's exact test, as appropriate. Correlation analyses were performed using Spearman's rank correlation. The statistically significant was determined with *p* Values less than 0.05, which was denoted as *p < 0.05, **p < 0.01, ***p < 0.001 and ****p < 0.0001, respectively.

## Author Contributions

**R.W**., and **M.L**. contributed equally to this work. Conceptualization was carried out by **Q.L**., **M.L**., and **R.W**. Methodology was dealt with by **R.W**., **C.G**., **C.Z**., **F.Z**., **S.D**., **B.Z**., **Q.L**., and **W.Z**. Investigation was performed by **R.W**., **C.G**., **C.Z**., **F.Z**., **S.D**., **B.Z**., **Q.L**., and **W.Z**. Visualization was carried out by **Q.L**., **R.W**., **M.Z**., and **M.L**. Funding acquisition was performed by **Q.L**., **M.Z**., and **M.L**. Project administration was carried out by **Q.L**., **M.Z**., and **M.L**. Supervision was performed by **Q.L**., **M.Z**., and **M.L**. Writing – original draft was carried out by **R.W**. Writing – review & editing was performed by **Q.L**., **M.Z**., and **M.L**.

## Conflicts of Interest

The authors declare no conflicts of interest.

## Supporting information




**Supporting File**: advs74453‐sup‐0001‐SuppMat.pdf

## Data Availability

The data that support the findings of this study are available from the corresponding author upon reasonable request.
