# Supplementary material for: Polydopamine‐Encapsulated Probiotics Restore Gut Homeostasis and Reinstate Macrophage Efferocytosis in Systemic Lupus Erythematosus
Source: Adv Sci (Weinh). 2026 Feb 20;13(24):e22637. doi: 10.1002/advs.202522637 (PMC13116111; doi:10.1002/advs.202522637)
Supplement: Supplementary file 1 — Supporting File: advs74453‐sup‐0001‐SuppMat.pdf [file ADVS-13-e22637-s001.docx]

**Polydopamine-Encapsulated Probiotics Restore Gut Homeostasis and Reinstate Macrophage Efferocytosis in Systemic Lupus Erythematosus**

Ruimiao Wu^1,2,3#^, Mei Liu^1,2,3*#^, Changxing Gao^1,2,3^, Cheng Zhao^4^, Feijie Zhao^5^, Shuqing Dong^1,2,3^, Bo Zhang^1,2,3^, Qianmei Liu^1,2,3^, Wenqian Zhang^6^, Ming Zhao^1,2,3,6*^, Qianjin Lu^1,2,3*^

**Author Affiliations:**

^1^ Hospital for Skin Diseases, Institute of Dermatology, Chinese Academy of Medical Sciences and Peking Union Medical College, Nanjing, 210042, China.

^2^ Key Laboratory of Basic and Translational Research on Immune-Mediated Skin Diseases, Chinese Academy of Medical Sciences, Nanjing, 210042, China.

^3^ Jiangsu Provincial Key Laboratory of Dermatology, Nanjing, 210042, China.

^4^ Shandong First Medical University, Shandong, 271016, China

^5^ Hunan University of Technology, Hunan, 411201, China

^6^ Department of Dermatology, Hunan Key Laboratory of Medical Epigenomics, the Second Xiangya Hospital, Central South University, Changsha, 410011, China

#These authors contributed equally to this work

***Correspondence Authors:**

Qianjin Lu, E-mail: [qianlu5860@pumcderm.cams.cn](mailto:qianlu5860@pumcderm.cams.cn)

Ming Zhao, E-mail: zhaoming301@pumcderm.cams.cn

Mei Liu, E-mail: pysliumei@pumcderm.cams.cn

**Supplemental Method**

**1. 16S rDNA Sequencing and Bioinformatic Analysis**

**1.1. DNA extraction and amplification**

Stool samples were snap-frozen immediately after collection and stored at −80 °C until use. Bacterial genomic DNA was extracted using the DNeasy PowerSoil Kit (Qiagen, Hilden, Germany). The DNA purity and integrity were assessed by NanoDrop 2000 spectrophotometry (Thermo Fisher Scientific, Waltham, MA, USA), and confirmed by agarose gel electrophoresis. The V3–V4 hypervariable regions of the bacterial 16S rRNA gene were amplified in 25 µL reactions using universal primers (343F: 5′-TACGGRAGGCAGCAG-3′; 798R: 5′-AGGGTATCTAATCCT-3′), with sample-specific barcodes incorporated into the reverse primer and Illumina adapters ligated to both ends.

**1.2. Library construction and sequencing**

Amplicons were visualized by agarose gel electrophoresis, purified with Agencourt AMPure XP beads (Beckman Coulter, USA), and quantified with the Qubit dsDNA Assay Kit. Equimolar amounts were pooled and sequenced on an Illumina NovaSeq 6000 platform (Illumina, San Diego, CA) with paired-end 250 bp reads (OE Biotech Co., Ltd., Shanghai, China).

**1.3. Bioinformatic analysis**

Raw FASTQ data were processed using the QIIME2 pipeline (version 2020.11). Adapter sequences were trimmed with Cutadapt, followed by quality filtering, denoising, merging of paired-end reads, and chimera removal using DADA2 with default parameters. Amplicon sequence variants (ASVs) were generated, and representative sequences were assigned taxonomies against the SILVA 138 database using the QIIME2 feature-classifier plugin. Alpha diversity indices (Chao1, Shannon, Observed_species, and Simpson) were calculated to estimate microbial richness and diversity. Beta diversity was assessed by weighted UniFrac distances, which were used for principal coordinates analysis (PCoA) and phylogenetic tree construction. The 16S rRNA gene amplicon sequencing and analysis were conducted by OE Biotech Co., Ltd. (Shanghai, China).

**2. Untargeted Metabolomics Analysis by LC–MS/MS of stool samples**

Metabolite profiling was performed by OE Biotech Co., Ltd. (Shanghai, China) using an ACQUITY UPLC I-Class Plus system (Waters Corporation, Milford, USA) coupled to a Q Exactive Plus mass spectrometer equipped with a heated electrospray ionization (HESI) source (Thermo Fisher Scientific, Waltham, MA, USA). Separation was achieved on an ACQUITY UPLC HSS T3 column (100 × 2.1 mm, 1.8 μm) under both positive and negative ion modes. The binary mobile phases consisted of water with 0.1% formic acid (A) and acetonitrile (B). The gradient was programmed as follows: 0.01 min, 5% B; 2min, 5% B; 4min, 30% B; 8min, 50% B; 10min, 80% B; 14min, 100% B; 15 min, 100% B; 15.1 min, 5% and 16 min, 5% B. The flow rate was 0.35 mL/min, column temperature 45 °C, and injection volume 2 μL. Samples were maintained at 10 °C throughout analysis.

The mass spectrometer was operated with the following settings: scan range m/z 100–1000, full MS resolution 70,000, HCD-MS/MS resolution 17,500, and stepped collision energies of 10, 20, and 40 eV. Additional parameters included spray voltage 3800 V (+) and 3200 V (−), sheath gas 35, auxiliary gas 8 (arbitrary units), capillary temperature 320 °C, auxiliary gas heater temperature 350 °C, and S-lens RF level 50.

The original LC-MS data were processed using XCMS v4.5.1 for peak detection, integration, retention time alignment, and normalization. Metabolite annotation was based on accurate mass, retention time, MS/MS fragmentation, and isotopic distribution, and cross-referenced against HMDB, LipidMaps (v2.3), METLIN, and LuMet-Animal 3.0 databases. Features with >50% missing values within a group, or with identification scores below 36 (out of 80), were excluded. Zero intensities were replaced with half of the minimum positive value, and data from both ionization modes were combined into a single matrix.

The matrix was imported in the statistical software R to carry out Principal Component Analysis (PCA) to observe the overall distribution among the samples and the stability of the whole analysis process. Orthogonal Partial Least-Squares-Discriminant Analysis (OPLS-DA) was utilized to distinguish the metabolites that differ between groups. To prevent overfitting, 7-fold cross-validation and 200 Response Permutation Testing (RPT) were used to evaluate the quality of the model. Variable Importance of Projection (VIP) values obtained from the OPLS-DA model were used to rank the overall contribution of each variable to group discrimination. A two-tailed Student’s T-test was further used to verify whether the metabolites of difference between groups were significant. Volcano plots were used to filter metabolites of interest which was based on log2 (Fold Change, FC) and ‐log10 (p‐value) of metabolites. Differential metabolites were selected with VIP values＞1.0, p-values＜0.05 and |log_2_FC|＞1, which were further used for KEGG pathway (http://www.genome.jp/kegg/) enrichment analysis.

**Supplemental Figures**


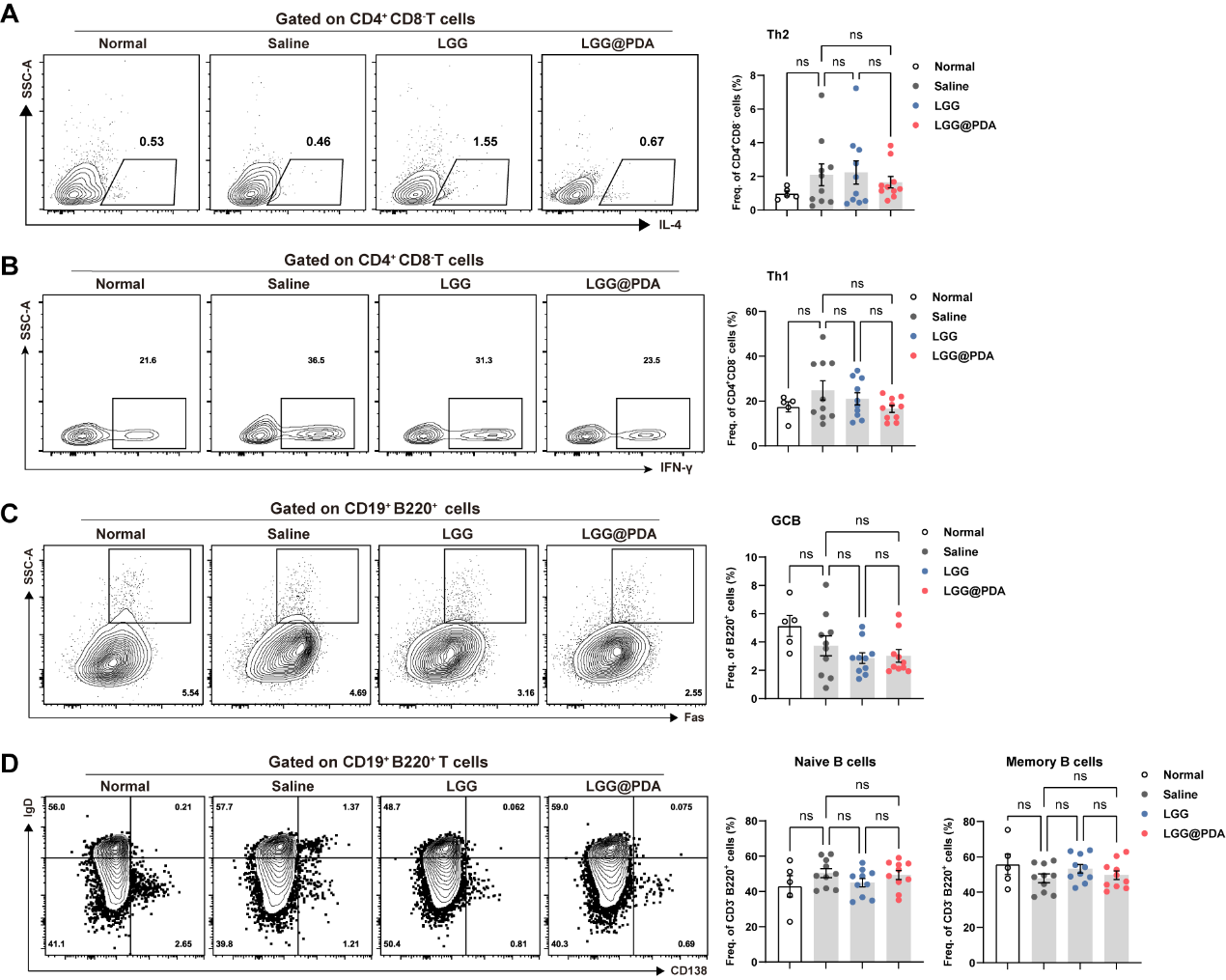


**Figure S1. Flow cytometric analysis of splenic lymphocyte subsets.** (A-B) Representative flow cytometry plots and quantitative summary of splenic CD4⁺ T cell subsets, showing the frequency of Th2 cells (A) and Th1 cells (B). (C-D) Representative plots and quantitative summary data of splenic B cell subsets, including germinal center B (GCB) cells (C), naïve B cells and memory B cells (D). Each point represents one subject, and bars indicate the mean ± SEM. Statistical significance was determined using one-way ANOVA (A-D).  ^∗^*p* < 0.05, ^∗∗^*p* < 0.01, ^∗∗∗^*p* < 0.001, ^∗∗∗∗^*p* < 0.0001; ns, not significant.


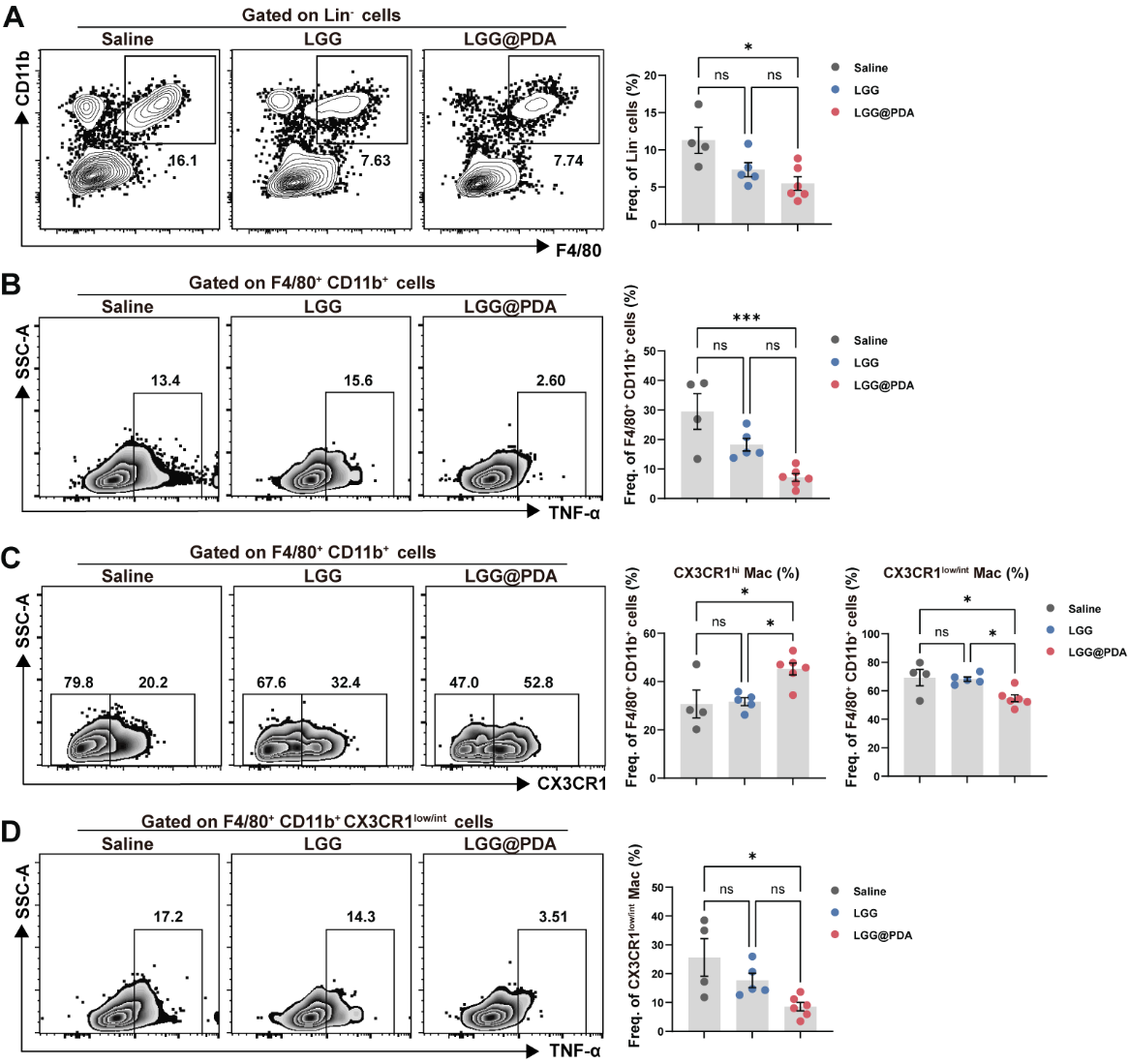


**Figure S2. Flow cytometric analysis of lamina propria macrophages.**

(A) Total macrophages (Zombie Aqua^-^CD45^+^Lin^-^F4/80^+^CD11b^+^). (B) TNF-α^+^ macrophages (Zombie Aqua^-^CD45^+^Lin^-^F4/80^+^CD11b^+^TNF-α^+^). (C) CX3CR1^low/int^ and CX3CR1^hi^ macrophage subsets (Zombie Aqua^-^CD45^+^Lin^-^F4/80^+^CD11b^+^CX3CR1^low/int/hi^). (D) TNF-α^+^ CX3CR1^low/int^ macrophages (Zombie Aqua^-^CD45^+^Lin^-^F4/80^+^CD11b^+^CX3CR1^low/int^TNF-α^+^). Each point represents one subject, and bars indicate the mean ± SEM. Statistical significance was determined using one-way ANOVA (A-D).  ^∗^*p* < 0.05, ^∗∗^*p* < 0.01, ^∗∗∗^*p* < 0.001, ^∗∗∗∗^*p* < 0.0001; ns, not significant.


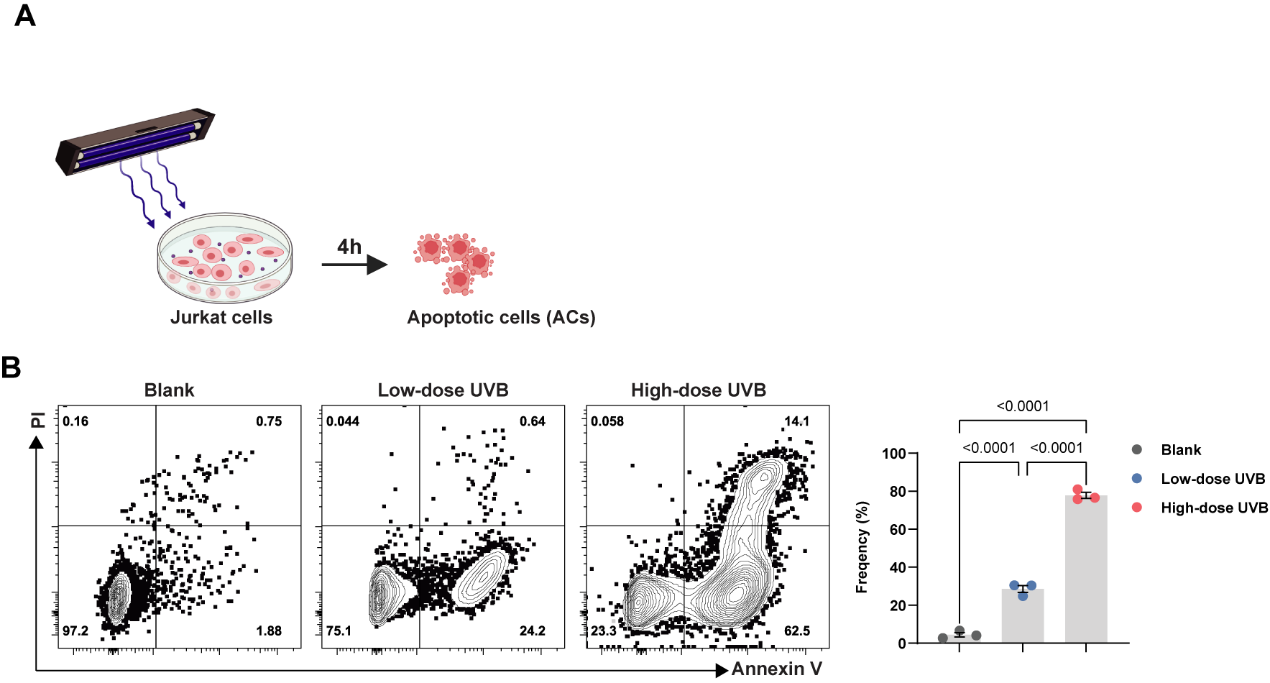


**Figure S3. UVB-induced apoptosis in Jurkat cells.** (A) Schematic illustration of the experimental procedure for UVB-induced apoptosis in Jurkat cells. (B) Representative flow cytometry plots and quantitative analysis of Jurkat cells under low-dose UVB (180 mJ/cm²) and high-dose UVB (360 mJ/cm²) exposure.

**Supplemental Table**

**Table S1. Differential metabolites associated with changes in clinical features, gut microbiota alterations, and KEGG pathway enrichment.**

|  | **Name** | **Superclass** | **VIP†** | **FC‡** | ***p*** | |
| --- | --- | --- | --- | --- | --- | --- |
| Metabolites associated with changes in clinical features | Scyllo-Inositol | Organic oxygen compounds | 1.28 | 2.00 | 0.00 | |
|  | Dihydroferulic acid | Phenylpropanoids and polyketides | 2.22 | 0.16 | 0.00 | |
|  | D-Phenylalanine | Organic acids and derivatives | 1.20 | 1.93 | 0.02 | |
|  | 2-Amino-5-ureidopentanoic acid | Organic acids and derivatives | 1.62 | 3.16 | 0.01 | |
|  | L-Methionine | Organic acids and derivatives | 1.34 | 2.25 | 0.02 | |
|  | DL-Proline | Organic acids and derivatives | 1.37 | 2.23 | 0.01 | |
|  | L-Alloisoleucine | Organic acids and derivatives | 1.14 | 1.89 | 0.03 | |
|  | DTrE | Lipids and lipid-like molecules | 1.57 | 2.55 | 0.00 | |
|  | 22:5(4Z,7Z,10Z,13Z,16Z) | Lipids and lipid-like molecules | 1.47 | 2.59 | 0.01 | |
|  | Daidzein | Phenylpropanoids and polyketides | 1.68 | 0.30 | 0.02 | |
|  | Quinaldine | Organoheterocyclic compounds | 1.49 | 2.64 | 0.01 | |
|  | Oxindole | Organoheterocyclic compounds | 1.79 | 4.39 | 0.02 | |
|  | 10-[3]-ladderane-decanoic acid | Lipids and lipid-like molecules | 1.24 | 1.80 | 0.00 | |
|  | D-(+)-Cellobiose | Organic oxygen compounds | 1.45 | 0.36 | 0.02 | |
|  | L-Leucine | Organic acids and derivatives | 1.14 | 1.89 | 0.03 | |
|  | 2,8-Quinolinediol 2-sulfate | Organoheterocyclic compounds | 1.45 | 2.70 | 0.02 | |
|  | Sphingosine | Organic nitrogen compounds | 1.64 | 3.07 | 0.01 | |
|  | Isoleucyl-Gamma-glutamate | Organic acids and derivatives | 1.54 | 2.71 | 0.01 | |
|  | Isotridecenoic acid | Lipids and lipid-like molecules | 1.78 | 3.39 | 0.00 | |
|  | L-Histidine | Organic acids and derivatives | 1.09 | 1.75 | 0.02 | |
|  | 11Z,13Z-Octadecadienal | Lipids and lipid-like molecules | 2.58 | 10.77 | 0.00 | |
|  | Pyroglutamylvaline | Organic acids and derivatives | 1.19 | 1.82 | 0.01 | |
| Metabolites associated with gut microbiota alterations | Niacin | Organoheterocyclic compounds | 1.41 | 2.34 | 0.01 | |
|  | L-Methionine | Organic acids and derivatives | 1.34 | 2.25 | 0.02 | |
|  | Imidazolepropionic acid | Organoheterocyclic compounds | 2.15 | 5.54 | 0.00 | |
|  | Ricinoleic Acid methyl ester | Lipids and lipid-like molecules | 1.69 | 3.23 | 0.01 | |
|  | ETrE(5Z, 8Z, 11Z) | Lipids and lipid-like molecules | 1.39 | 2.15 | 0.01 | |
|  | Sphingosine | Organic nitrogen compounds | 1.64 | 3.07 | 0.01 | |
| Metabolites associated with KEGG pathway enrichment (Cysteine/methionine metabolism^a^ and Efferocytosis^b^) | L-Methionine^ab^ | Organic acids and derivatives | 1.34 | 2.25 | 0.02 | |
|  | L-2-Aminobutyric acid^a^ | Organic acids and derivatives | 1.45 | 2.67 | 0.03 | |
|  | Cholesterol(d7)^b^ | Lipids and lipid-like molecules | 1.26 | 2.03 | 0.01 | |
|  | L-Cysteinesulfinic acid^a^ | Organic acids and derivatives | 1.63 | 3.06 | 0.01 | |
|  | L-Cysteine^a^ | Organic acids and derivatives | 2.15 | 8.19 | 0.02 | |
|  | Cysteic acid^a^ | Organic acids and derivatives | 1.59 | 2.93 | 0.01 | |
| Note: VIP > 1, \|log_2_FC\| > 1, p < 0.05. †VIP: variable importance for the projection. ‡ FC: fold change.  a: Metabolites enriched exclusively in Cysteine/methionine metabolism b: Metabolites enriched exclusively in Efferocytosis ab: Metabolites enriched in both Cysteine/methionine metabolism and Efferocytosis | | | | | |  |

**Table S2. List of primers used in this study.**

| **Gene** | **Forward primer (5' to 3')** | **Reverse primer (5' to 3')** |
| --- | --- | --- |
| *16SrRNA* | CGGGACACGAGCGCAACCC | CCATTGTAGCACGTGTCTAGCC |
| *LGG* | CGCCCTTAACAGCAGTCTTC | GCCCTCCGTATGCTTAAACC |
| *ZO-1* | GTTGGTACGGTGCCCTGAAAGA | GCTGACAGGTAGGACAGACGAT |
| *Claudin-2* | CAACTGGTGGGCTACATCCTA | CCCTTGGAAAAGCCAACCG |
| *Claudin-4* | GTCCTGGGAATCTCCTTGGC | TCTGTGCCGTGACGATGTTG |
| *Claudin-23* | CCCGACGAGTGGAACTACTTC | GGCCAGCGACGAAAAACAC |
| *Occludin* | TTGAAAGTCCACCTCCTTACAGA | CCGGATAAAAAGAGTACGCTGG |
| *CX3CR1* | GAGCATCACTGACATCTACCTCC | AGAAGGCAGTCGTGAGCTTGCA |
| *Mertk* | ATCATCCTCGGCTGCTTCTGTG | ACGACCAGTTGGGAATCCTCCT |
| *ProS* | TGGCAAGGAGACAGGTGTCAGT | GAGCAGTGGTAACTTCCAGGAG |
| *ITGAV* | GTGTGAGGAACTGGTCGCCTAT | CCGTTCTCTGGTCCAACCGATA |
| *MFG-E8* | GAGCAACAGTGCCAAGGAATGG | ACTGTGGGCTACCTTGTAGGAC |
| *CD300f* | GATGCTGGCATTTACTGGTGTGG | GGTTGTCACTGTGAAGATGGTGG |
| *TIM-4* | AGAATGTGCGCTTGGAGCTGAG | GGTTGGGAGAACAGATGTGGTGTC |
| *Gas6* | GAACTTGCCAGGCTCCTACTCT | GGAGTTGACACAGGTCTGCTCA |
| *RAGE* | GCCACTGGAATTGTCGATGAGG | GCTGTGAGTTCAGAGGCAGGAT |
| *GAPDH* | CATCACTGCCACCCAGAAGACTG | ATGCCAGTGAGCTTCCCGTTCAG |
| *β-actin* | AGCCATGTACGTAGCCATCC | CTCTCAGCTGTGGTGGTGAA |

**Table S3. List of antibodies used in this study.**

| **Antibodies** | **Source** | **Identifier** |
| --- | --- | --- |
| Zombie Aqua™ Fixable Viability Kit | Biolegend | 423102 |
| APC-Cy™7 Rat Anti-Mouse CD3 Molecular Complex | BD | 560590 |
| PE anti-mouse CD3 Antibody | Biolegend | 100206 |
| Alexa Fluor® 700 Rat Anti-Mouse CD4 | BD | 557956 |
| BV605 Rat Anti-Mouse CD8a | BD | 563152 |
| APC anti-mouse IFN-γ Antibody | Biolegend | 505810 |
| PE-Cy™7 Rat Anti-Mouse IL-4 | BD | 560699 |
| IL-17A Monoclonal Antibody (eBio17B7), eFluor™ 450 | Invitrogen | 48-7177-82 |
| CD25 Monoclonal Antibody (PC61.5), Super Bright™ 780 | Invitrogen | 78-0251-82 |
| Alexa Fluor® 488 anti-mouse/rat/human FOXP3 Antibody | Biolegend | 320012 |
| BB700 Hamster Anti-Mouse CD279 (PD-1) | BD | 566514 |
| Biotin Rat Anti-Mouse CD185 (CXCR5) | BD | 551960 |
| PE Streptavidin | BD | 554061 |
| mouse Fc‐R block | Biolegend | 101302 |
| Brilliant Violet 711™ anti-mouse CD19 Antibody | Biolegend | 115555 |
| PE/Cyanine7 anti-mouse/human CD45R/B220 Antibody | Biolegend | 103222 |
| Brilliant Violet 421™ anti-mouse CD138 (Syndecan-1) Antibody | Biolegend | 142508 |
| PerCP-Cy™5.5 Rat Anti-Mouse IgD | BD | 564273 |
| FITC anti-mouse/human CD44 Antibody | Biolegend | 103006 |
| Alexa Fluor® 647 Rat Anti-Mouse GL-7 | BD | 561529 |
| APC/Fire™ 810 anti-mouse CD95 (Fas) Antibody | Biolegend | 152624 |
| Brilliant Violet 650™ anti-mouse F4/80 Antibody | Biolegend | 123149 |
| Brilliant Violet 421™ anti-mouse CD86 Antibody | Biolegend | 105032 |
| APC anti-mouse/human CD11b Antibody | Biolegend | 101212 |
| PE anti-mouse CD206 (MMR) Antibody | Biolegend | 141706 |
| Alexa Fluor® 700 Rat Anti-Mouse CD45 | BD | 560510 |
| FITC anti-mouse Ly-6G Antibody | Biolegend | 127606 |
| Alexa Fluor® 594 anti-mouse CD45.2 Antibody | Biolegend | 109850 |
| APC/Cyanine7 anti-mouse CD4 Antibody | Biolegend | 100414 |
| Alexa Fluor® 700 anti-mouse CD25 Antibody | Biolegend | 102024 |
| PE/Cyanine7 anti-mouse F4/80 Antibody | Biolegend | 123113 |
| Brilliant Violet 605™ anti-mouse/human CD11b Antibody | Biolegend | 101257 |
| Alexa Fluor™ 647 Rat Anti-Mouse CX3CR1 | BD | 567805 |
| Brilliant Violet 750™ anti-mouse TNF-α Antibody | Biolegend | 506358 |
| PerCP/Cyanine5.5 anti-mouse CD45.2 Antibody | Biolegend | 109828 |
| FITC anti-mouse/human CD11b Antibody | Biolegend | 101206 |
| PerCP-Cy™5.5 Mouse Lineage Antibody Cocktail | BD | 561317 |
| PE anti-human CX3CR1 Antibody | Biolegend | 341603 |
| APC/Cyanine7 anti-human CD68 Antibody | Biolegend | 333821 |
| FITC anti-human CD45 Antibody | Biolegend | 304006 |
